# Structural insights into the substrate binding mechanism of the class I dehydratase MadB

**DOI:** 10.1038/s42003-025-08454-5

**Published:** 2025-07-09

**Authors:** C. Vivien Knospe, Julio Ortiz, Jens Reiners, Alexej Kedrov, Christoph G. W. Gertzen, Sander H. J. Smits, Lutz Schmitt

**Affiliations:** 1 Institute of Biochemistry, Heinrich Heine University Düsseldorf, Düsseldorf, Germany; 2 Ernst Ruska-Centre for Microscopy and Spectroscopy with Electrons, Forschungszentrum Jülich, Jülich, Germany; 3 Center for Structural Studies, Heinrich Heine University Düsseldorf, Düsseldorf, Germany; 4 Synthetic Membrane Systems, Institute of Biochemistry, Heinrich Heine University Düsseldorf, Düsseldorf, Germany; 5 Present Address: Thermo Fisher Scientific, Materials & Structural Analysis, Dreieich, Germany

**Keywords:** Cryoelectron microscopy, Proteins

## Abstract

In the battle against antimicrobial resistance, lantibiotics have emerged as promising new sources for antimicrobial drugs. Their exceptional stability is due to characteristic modifications termed (methyl-)lanthionine rings. Genome mining efforts have identified hundreds of lantibiotics by detecting gene operons, so-called biosynthetic gene clusters (BGC), which encode cysteine-rich peptides (30-50 amino acids in size) and enzymes responsible for dehydration and cyclization, catalyzing the post-translational ring formation. One such identified, class I lantibiotic is maddinglicin from *Clostridium maddingley*. Here, we present single particle cryo-EM structures of the dehydratase MadB in both, its apo-state and in complex with a leader peptide of maddinglicin, revealing a distinct conformational change upon substrate binding. Small-angle X-ray scattering studies elucidate the substrate binding site for the C-terminal part of maddinglicin. Furthermore, a substrate specificity analysis was performed highlighting a critical stretch of amino acids within the maddinglicin leader sequence that is crucial for binding. Here, we provide molecular insights into the conformational changes, principles of substrate recognition and ligand:protein stoichiometry of a class I lantibiotic dehydratase.

## Abstract (web summary)

Class I lantibiotic dehydratases play a key role in the biosynthesis of ribosomally synthesized peptide antibiotics. We present structural insights into MadB in two states, revealing the conformational changes that underlie its catalytic mechanism during maddinglicin maturation.

## Introduction

Maddinglicin A (MadA) produced by *Clostridum maddingley* belongs to a group of antimicrobial peptides, the so-called lantibiotics or in brief LanA^1^. These form the largest sub-family of ribosomally synthesized and post-translationally modified (PTM) peptides (RiPPs), which display diverse bioactivities (e.g., antimicrobial, antiviral, or antifungal)^2,3^ and are of high interest in the battle against bacterial infections. In general, they consist of an N-terminal leader peptide (LP), which is important for the recognition by the PTM enzymes^4–7^, transport^8^, and maintaining the inactive state of the lantibiotic within the cell^7^, and a C-terminal core peptide (CP), in which the PTMs are installed. The main characteristic of the CP is the presence of (methyl-)lanthionine rings generating higher stability and resistance against proteolysis, which raises interest in this class of compounds as potential new sources for antibiotics^9–12^.

MadA was initially characterized by van Heel et al.^1^, who used the nisin modification machinery to discover and characterize novel lantibiotics from genome mining approaches. Among the five active lantibiotics that were identified out of 54 candidate lantibiotics, MadA possesses the largest deviation in amino acid sequence compared to NisA. The architecture of the MadA operon resembles the general features of class I lantibiotic operons as it contains genes encoding for a dehydratase (in general called LanB, in the Mad system called MadB) and a cyclase (in general called LanC, in the Mad system called MadC) next to MadA. The paradigm of class I lantibiotics is nisin or NisA from *Lactococcus lactis*^13,14^. It contains five (methyl-)lanthionine rings and a hinge region between the third and fourth ring. This hinge region is essential for membrane insertion of the last two C-terminal rings, which follows the initial formation of a lipid II complex by the three N-terminal rings^15^. Thus, nisin possesses both, bacteriostatic (preventing cell wall formation by interaction with lipid II) and bactericidal (pore formation through membrane insertion) properties. MadA is predicted to contain seven (methyl-)lanthionine rings, of which the four C-terminal rings are highly intertwined^16,17^. A comparison of MadA and NisA revealed a high similarity of the three N-terminal rings with respect to position and size. Obviously, one can assume that the function of the first two N-terminal rings (binding to lipid II) is conserved between the two lantibiotics. On the other hand, MadA lacks the hinge region of NisA and the numbers of (methyl)lanthionine rings in the C-terminal part is different as NisA contains two but MadA four rings. This suggests that MadA lacks a nisin-like pore-forming activity.

The PTMs resulting in the formation of (methyl-)lanthionine rings are exclusively installed in the CP. The first necessary step is the dehydration of serine and threonine residues, which is catalyzed by the MadB enzyme in the case of MadA. The second step represents the condensation of these dehydrated residues with a neighboring cysteine side chain resulting in (methyl-)lanthionine rings, which is guided by the MadC enzyme in the case of MadA^18^. Importantly, all so far analyzed LanBs of class I lantibiotics catalyze the dehydration reaction via a glutamylated intermediate in a tRNA^Glu^-dependent manner, while LanC enzymes are Zn^2+^-dependent enzymes^19^.

LanBs contain a structural feature called the RiPP recognition element (RRE), which seems essential for binding of the LP of LanAs to the dehydratase^20^. It is known that LanBs are composed of a glutamylation and elimination domain based on the crystal structures of NisB, the dehydratase of the lantibiotic nisin, and MibB, the dehydratase from *Microbispora sp*. 107891, which both function as homo-dimers^5,21–23^. MadB shares 26.8% sequence identity with NisB and 24.2% with MibB. In addition, MadB with 1030 amino acids is in between NisB (993 amino acids) and MibB (1115 amino acids). The latter possesses an extended N-terminus. Furthermore, the dehydration reaction is not only tRNA^Glu^ -dependent, but it also displays tRNA specificity, which can be organism- or isoacceptor-specific^21,22^. Even though MibB^21^ and TbtB^24^ are examples for tRNA-specificity, NisB^22^ is clearly promiscuous and the *E. coli* tRNA^Glu^ can serve as a surrogate. This raises the question which region(s) of the dehydratase is (are) responsible for this tRNA^Glu^ specificity. So far however, conformational changes of a class I dehydratase could only be derived from different enzyme structures, i.e., the NisB:NisA complex and the apo-state of MibB. The above-described differences between NisA and MadA motivated us to characterize MadB, the dehydratase of MadA, in more detail as these functional differences might be reflected by structural differences between the three dehydratases.

Here, we present an in-depth characterization of MadB, the dehydratase of MadA from *C. maddingley*, including functional data as well as the single particle cryo-EM structures of the apo- and ligand-bound state. These results provide new insights into substrate-induced conformational changes, substrate specificity, and the in vitro stoichiometry of the MadB-MadA complex.

## Results

**Fig. 1:**
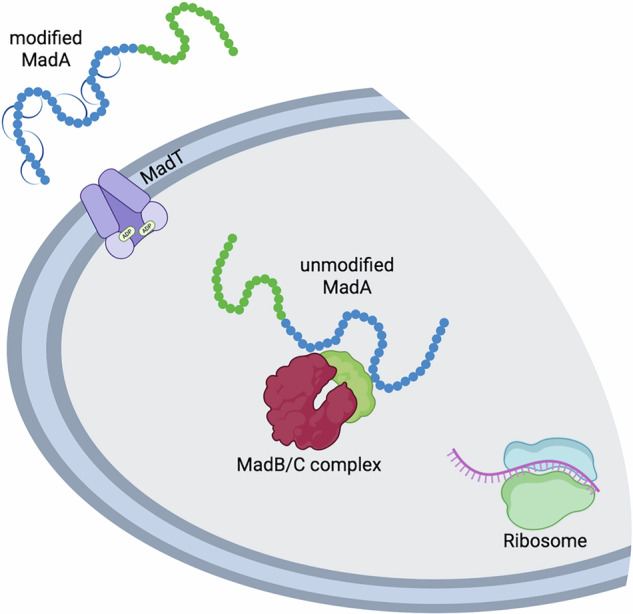
Schematic overview of the MadA maturation and secretion system. The figure has been created using BioRender with permission (Created in BioRender. Schmitt, L. (2025) https://BioRender.com/6e32ynv).

The BGC of MadA resembles the classic architecture of a class I lantibiotic operon. After ribosomal synthesis, the fully unmodified MadA is dehydrated and cyclized by the maturation enzymes MadB and MadC, respectively. The LP is responsible for binding to the two enzymes that install the PTMs solely in the CP. However, the directionality and the exact mechanism is currently unknown. After the PTMs have been introduced completely in the CP, fully modified MadA is secreted by the ABC transporter MadT (Fig. 1) and subsequently activated by removal of the LP (for reviews see refs. ^13,19,25^).

### Purification of His_6_-MadB

**Fig. 2:**
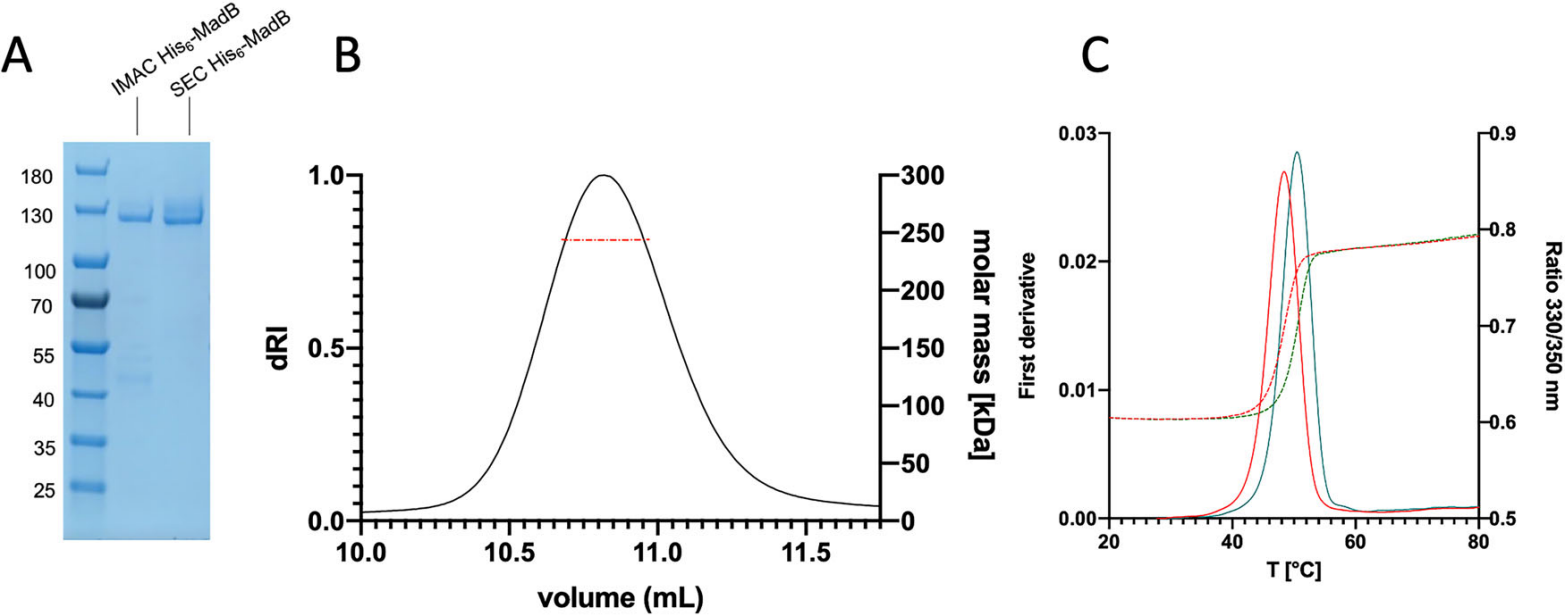
Expression and characterization of MadB. **A** Coomassie brilliant blue stained SDS PAGE of purified MadB after IMAC and SEC. Molecular weights of marker proteins are given to the left in kDa. The uncropped gel is shown in Fig. S9. **B** SEC-MALS analysis of MadB after SEC purification. Plotted is the RI value of the elution profile. The calculated molecular weight of the sample is indicated by the red horizontal line. **C** NanoDSF analysis of MadB (red) and MadB incubated with MadL1 (green).

For a detailed structure-function relation analysis of MadB, the dehydratase was expressed in *E. coli* BL21(DE3) and purified to homogeneity using IMAC and SEC (Fig. 2A) as described in “Materials and methods”. The yield was ~7 mg of purified protein from 2 L expression culture. Additionally, SEC–multi-angle light scattering (MALS) experiments (Fig. 2B) revealed a molecular mass of 243.6 kDa (±0.2 kDa), which is close to the theoretical molecular weight of a MadB dimer based on the sequence (249.7 kDa for dimeric MadB), demonstrating that MadB forms a dimer in solution, similar to NisB^5^.

### Analysis of the MadA : MadB complex

**Fig. 3:**
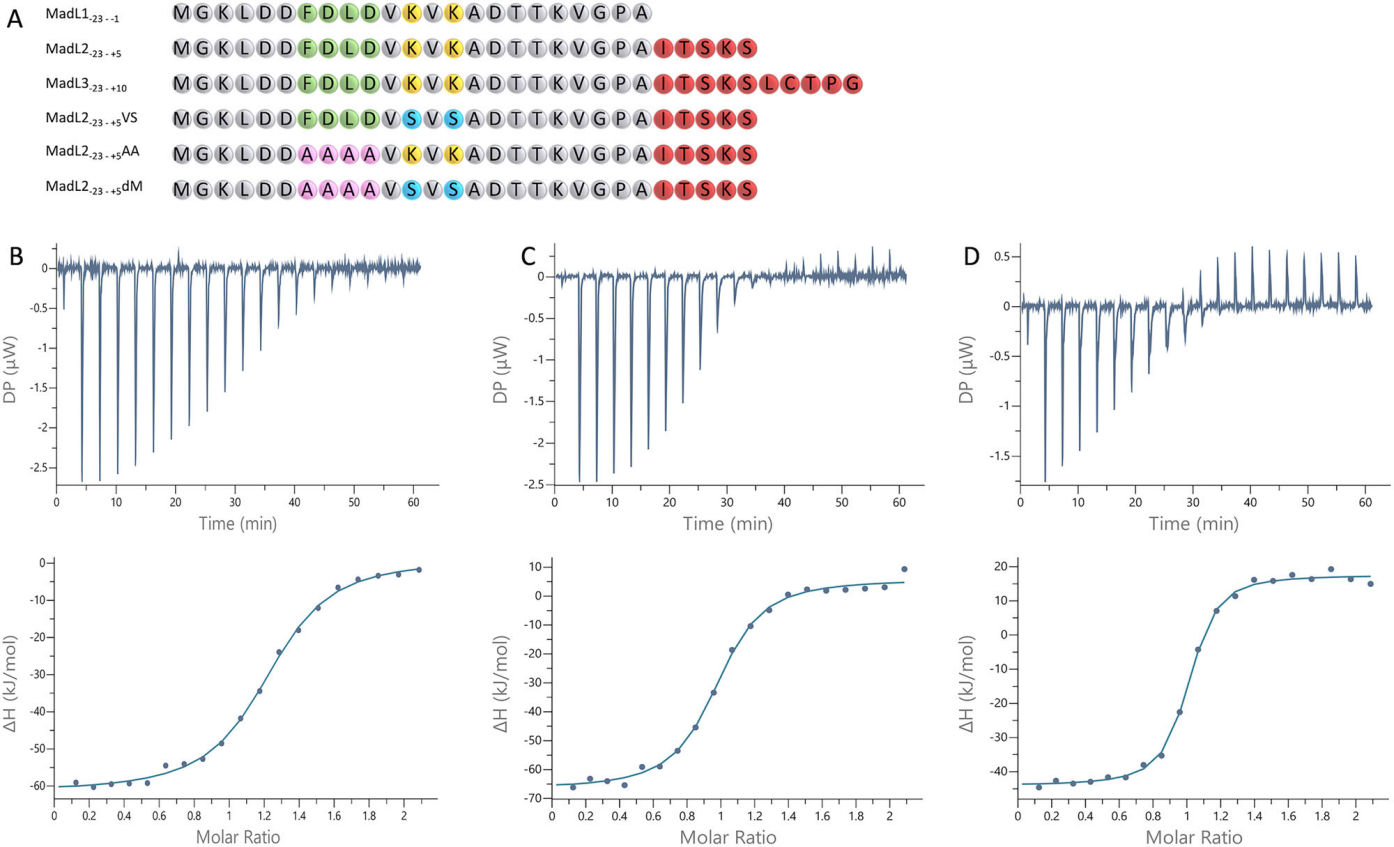
ITC analysis of different LP variants to MadB. **A** Schematic summary of the MadA variants used in this study. Residues of the CP are highlighted in red, the FDLD box of MadB in green and the corresponding AAAA mutation in magenta. The two lysine residues of the VKVK motive are shown in yellow and the mutations to serine residues in blue. ITC raw data (upper panels) and analyzed data (lower panels) are shown for MadL1/MadB (**B**), MadL2/MadB (**C**) and MadL3/MadB (**D**).

Nano differential scanning fluorimetry (DSF) was employed to probe a possible complex formation between MadA and MadB (Fig. 2C and Table S1). MadB underwent a cooperative thermal denaturation at 48.4 °C (melting temperature, *T*_m_) in its apo-state. Upon addition of the isolated LP (MadL1), a shift of +1.8 °C was evident suggesting that the protein was stabilized upon binding the substrate mimetic. In addition to MadL1 (LP) we synthesized variants that covered amino acids of the N-terminal part of the CP (Fig. 3A). The variant MadL2 contained the first five N-terminal amino acids of the CP including Thr^2^ and Ser^5^, which likely become dehydrated, and Ser^3^, while ten N-terminal amino acids of the CP including additionally Cys^7^ and Thr^8^ were present in MadL3 (Fig. 3A). Increasing the CP part of the construct led to higher T_m_ and consequently to a higher protein stabilization by complex formation (Table S1).

To obtain in-depth quantitative information on complex formation, we turned to isothermal titration calorimetry (ITC) to determine the thermodynamic parameters including the stoichiometry. For a detailed description of the experimental set-up, see “Materials and methods”. Titrating each of these three constructs (MadL1, MadL2, and MadL3) to MadB resulted in an isotherm indicating specific binding (Fig. 3B–D). The determined dissociation constants (*K*_D_) of 0.41 ± 0.04 µM (MadL1), 0.32 ± 0.04 µM (MadL2), and 0.15 ± 0.01 µM (MadL3) suggested that a prolongation of the LP with amino acids of the CP increased the affinity to MadB moderately (Table S2). The LP:MadB dimer stoichiometry of 1:1 was identical within experimental error for all three LP variants, being in agreement with the results obtained for the NisBA^5^ and NisBCA complexes^23^. The LP seems to be sufficient for substrate recognition, but the CP, in which the modifications take place, also affects binding affinity albeit in a smaller manner.

### Single particle cryo-EM structures of the apo and ligand-bound states of MadB

**Fig. 4:**
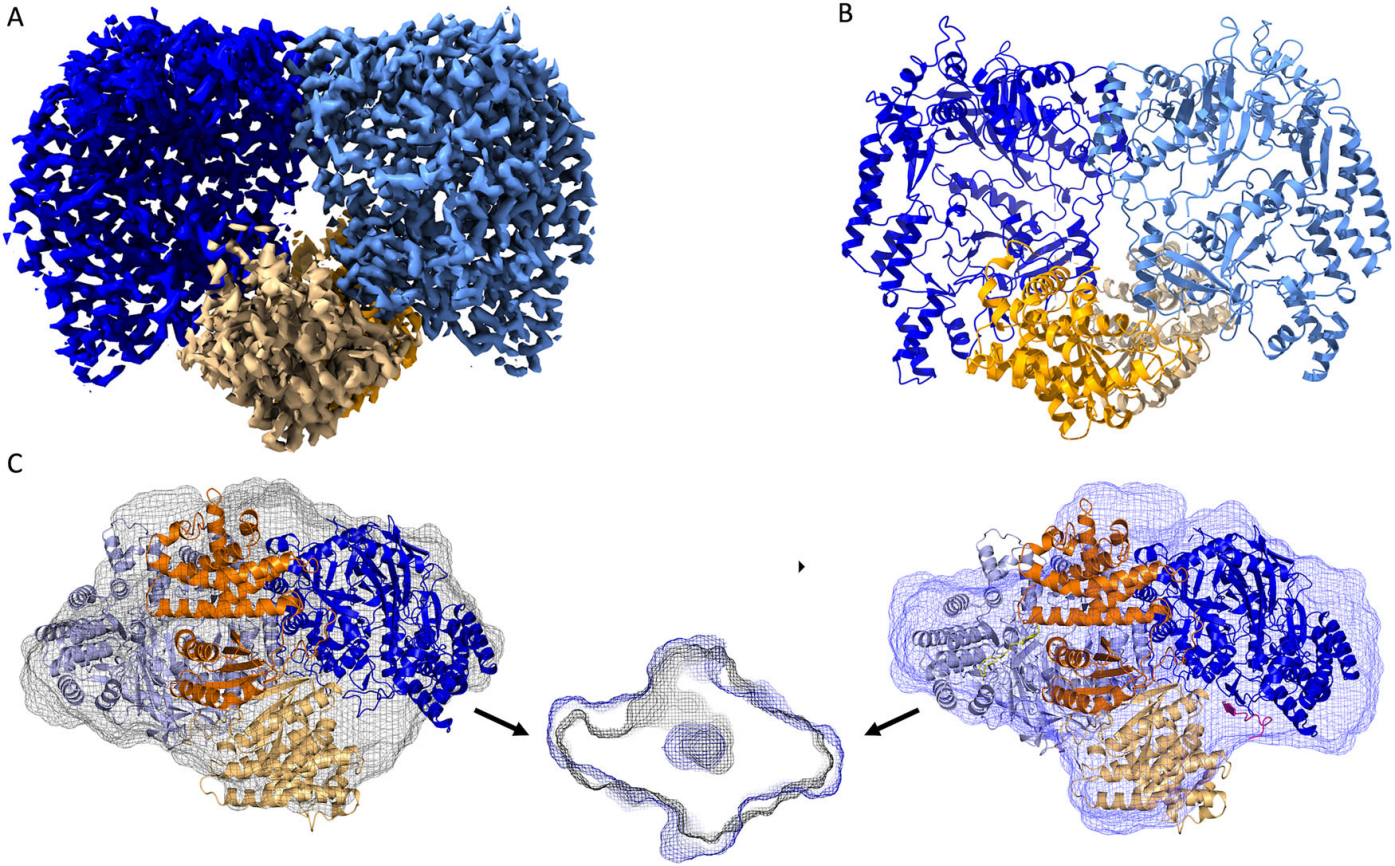
Structure of apo MadB. **A** Density of apo-MadB at an overall resolution of 2.7 Å. Color coding is identical to (**B**). **B** Cartoon representation of the overall structure of apo-MadB. The glutamylation domains are colored blue (GD1) and light blue (GD2) and the elimination domains in orange (ED1) and in light orange (ED2), respectively. **C** Docking of the apo-structure of MadB (left panel) into the apo-MadB SAXS envelope (gray) and MadB-MadL3 into the corresponding MadB-MadL3 SAXS envelope (blue, right panel). Color coding is identical to (**A**). Lower panel: overlay of the two SAXS envelops highlighting the presence of one tunnel in the apo structure of MadB leading to the RRE, which is absent in the complex with MadL3.

In order to obtain structural insights into the architecture of MadB and the MadA / MadB complex, we used single-particle cryo-EM. The structure of MadB in the apo-state was determined at an overall resolution of 2.7 Å (Fig. 4). MadB is composed of an N-terminal glutamylation (residues 1–741) and a C-terminal elimination (residues 742–1038) domain (see Fig. S1 for the sequence). MadB forms a homodimer as already demonstrated by SEC-MALS (Fig. 2B) with a bifurcate cleft-like structure in the center. The first and last amino acids of each monomer, residues 741–753 as well as 928–946 of elimination domain 1 (ED1: colored orange in Fig. 2A) and 930–936 of ED2 (colored light orange in Fig. 4B) were not modeled due to a lack of electron density. Comparison with the class I dehydratase MibB resulted in a root mean square deviation (RMSD) of 4.6 Å for 1727 Cα atoms indicating a low degree of similarity even though the overall structure visually seems to be very similar. Similarly, for NisB a RMSD of 2.6 Å for 1383 Cα atoms was determined. This indicates that the structure of apo MadB adopts a conformation in between the apo structure of MibB and the substrate-bound structure of NisB. Sequence alignments also revealed that all amino acids previously highlighted to be essential for the glutamylation (Arg^89^, Arg^93^, Thr^95^, Asp^128^, Asp^308^, Arg^483^) and elimination (Arg^822^, Arg^864^, His^1005^) reactions of NisB are conserved in MadB (highlighted in bold and underlined in Fig. S1 and highlighted in ball-and-sticks representation in Fig. S2)^26^.

**Fig. 5:**
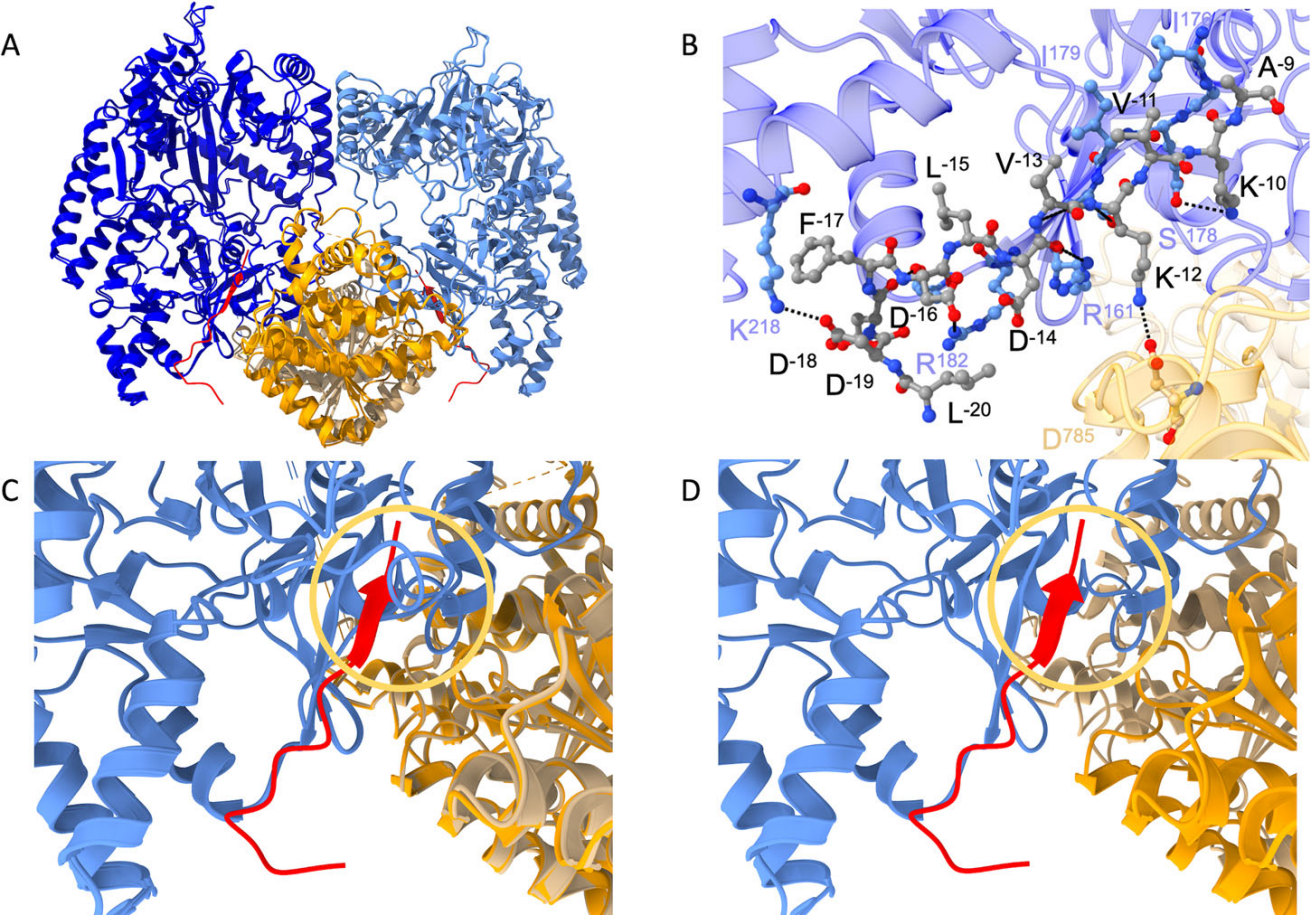
Structure of ligand-bound MadB. **A** Cartoon representation of the overall structure of MadB in complex with MadL3. Color-coding is identical to Fig. 2. The part of MadL3 that could be traced in the density (Leu^−20^ to Ala^−9^) is highlighted in red. **B** Zoom-in into the MadB- MadL3 binding interface. The LP is shown in ball-and-sticks presentation (colored by element) and residues are labeled in black. H binds between residues of MadB and MadL3 are indicated by black, dashed lines, and the participating residues of MadB are labeled in blue. **C** Docking of MadL3 into the apo-structure of MadB highlighting a steric clashes occurring in the RRE. This region is indicated by the yellow circle. **D** Same as in (**C**), but MadL3 (red cartoon) bound to the liganded structure of MadB. Again, this region is indicated by the yellow circle. For a better visualization of the conformational changes accompanying ligand binding see Movies S1 and S2.

To elucidate conformational changes upon substrate binding, we also determined the structure of MadB in complex with the MadA variant MadL3 (Fig. 5A) at an overall resolution of 3.1 Å. MadL3 compromises the LP of MadA including the first ten N-terminal amino acids of the CP. Residues 742–750 (the first nine amino acid residues of the EDs) were not modeled due to a lack of electron density in each monomer. The LP of MadA binds to the RRE element in MadB (Fig. 5B), which is present in the majority of prokaryotic RiPP classes^20^. Substrate binding induced the formation of a four-stranded, anti-parallel β-sheet, which is a three-stranded, anti-parallel β-sheet in the apo-state (Fig. 5C, D). A comparison with the co-crystal structure of the NisB:NisA complex^22^ revealed a RMSD of 2.4 Å for 1361 Cα atom. Surprisingly, the comparison of the apo- and bound-state of MadB revealed a RMSD of 0.2 Å for 1792 Cα atom. This clearly demonstrates that hardly any conformational change occurs upon substrate binding. The only change occurred in the vicinity of the substrate binding site. More precisely, a loop formed by residues 164–179 had to rotate by 8° to free space for substrate binding, while other minor conformational changes of MadB were located in surface-exposed areas of the protein (see Fig. 5 as well as Movies S1 and S2) and are likely not relevant for catalysis. In the apo state, the regulatory loop is stabilized by hydrophobic interactions with Ser^154^, Glu^230^, Tyr^518^, Ser^521^, and Phe^542^ as well as hydrogen bonds between the hydroxyl group of Ser^180^ and Glu^178^ (distance of 2.7 Å) and between the side chain and backbone of Arg^229^ with the backbone of Thr^168^ (distance of 3 Å) and the side chain of Thr^168^ (distance of 2.7 Å). In the case of the ligand-bound state, hydrophobic interactions are formed with Ser^154^, Ile^155^, Arg^229^, Tyr^518^, Phe^542^, Leu^519^, Pro^592^, and Ile^595^ as well as Val^−5^, Lys^−6^, Val^−7^ and Ala^−9^ of the LP. Also, the number of hydrogen bonds increased. Now interactions are formed between the backbone of Lys^−8^ of the LP and the backbone of Glu^178^ (distance of 2.6 Å), the side chain of Lys^137^ and the side chain of Asp^169^ (distance of 2.9 Å), backbone of Ser^180^ and backbone of Leu^164^ (distance of 2.6 Å), side chain of Glu^230^ and the side chain of Thr^168^ (distance of 3 Å), and the side chain of Ser^521^ and the side chain of Lys^170^ (distance of 3.1 Å). The increased number of hydrophobic (5–12) and hydrogen bonds (3–5) indicates that the conformation of the regulatory loop in the ligand-bound state is energetically more stabilized. In addition, the bound ligand contributes in this stabilization through hydrophobic interactions and an H bond.

Using the extended version of a maddinglicin LP (MadL3) we successfully resolved the electron density for sub-set of the amino acids of the LP. Electron density for the stretch of residues ^−20^LDDFDLDVKVKA^−9^ was clearly resolved and could be built (see Movie S3). We investigated the interactions of MadB to its substrate via LigPlot+^27^ with standard settings, which mostly found backbone to backbone or residue to backbone hydrogen interactions (MadB-MadA: Thr^184^ – Asp^−18^; Gln^225^ – Leu^−15^; Arg^182^ – Asp^−19^ and Asp^−16^ ; Arg^161^ – Asp^−14^; Ser^180^ – Lys^−12^ ; Gly^177^ – Lys^−10^), but also some side chain to side chain interactions were observed (MadB-MadA: Lys^218^ – Asp^−19^; Glu^178^ – Lys^−10^; Asp^785^ – Lys^−12^) (see Fig. 5B). Beside hydrogen bond interactions also hydrophobic interactions could be observed for the amino acids F^−17^, L^−15^, V^−13^, V^−11^ of the MadA LP with the RRE of MadB (I^176^, I^179^, I^181^, P^186^, F^187^, I^213^, I^219^, F^222^). Interestingly, most interactions originate from amino acids of the GD, but also interactions from the ED of the opposing monomer towards the substrate were observed (e.g., Asp^785^). Visualizing these interactions in a more schematic graphic (see Fig. S3) reveals that the binding of the LP of MadA has a strongly hydrophobic interaction area and a hydrophilic interaction area on the opposite side.

The MadB:MadL3 complex reveals binding for two substrate-binding sites, but due to the lack of electron density only the amino acids from −20 to −9 of the substrate were modeled. However, it needs to be considered that we used a C2 symmetry for structure refinement in order to obtain a high-resolution structure. All attempts to avoid symmetry imposition during structure refinement resulted in a loss of resolution and could not further be pursued. Therefore, it is feasible that the occurrence of two MadA peptides in the MadB dimer is due to the imposed symmetry. Crystal structures of LanB so far highlight the occurrence of two substrates in the dimer^22,24^, but in vitro studies imply that only one substrate is bound to a LanB dimer (e.g., NisB - SPR studies^5^ and SEC-MALS complex studies^23^), which is in line with our ITC results for MadB.

The CP of MadA would be located in a cavity between GD and ED (see Fig. 3F) if one assumes a simple prolongation of the LP. For further investigations, we performed SAXS measurements of apo MadB (Fig. 4C) and co-incubated MadB with the maddinglicin variant MadL3 (Fig. 4C). Further details are provided in SI (Figs. S4 and S5 as well as Table 1). The determined surface of MadB in solution deviated from a fully symmetrical form, as expected for a low-resolution map. Nonetheless, the fitting of our structure into the SAXS data was satisfactory (Fig. 4C). Interestingly, only at one side of the apo-MadB dimer a tunnel of the GDs envelope was evident (Fig. 4C). The tunnel is protruding towards the corresponding ED of a monomer indicating that space is present for substrate binding. A similar opening could not be observed for the opposite side, which raises interest as to why only one side shows an opening. Interestingly, after adding MadL3 to MadB the tunnel disappears in the SAXS measurement (Fig. 4C). These results indicate that the substrate or maybe even more precisely the CP fills the previously observed gap and consequently closes the channel. The previous assumption that MadB binds only one substrate per functional dimer is supported by these SAXS results.

**Table 1:** Overall SAXS data

SAXS device	P12, PETRA III, DESY Hamburg^1^	BM29, ESRF Grenoble^2,3^
Data collection parameters		
Detector	PILATUS 6 M	PILATUS 2 M
Detector distance (m)	3.0	2.827
Beam size (µm × µm)	120 × 200	200 × 200
Wavelength (nm)	0.124	0.099
Sample environment	Quartz capillary, 1 mm ø	Quartz capillary,1 mm ø
*s* range (nm^−1^)^‡^	0.02–7.0	0.025–6.0
Absolute scaling method	Comparison with scattering from pure H_2_O	
Normalization	To transmit intensity by the beam-stop counter	
Scattering intensity scale	Absolute scale, cm^−1^	
Sample	apo MadB	MadB with MadL3
Organism	*Clostridium sp. Maddingley*	
UniProt ID	K6TUQ9 (MadB), K6SWQ2 (MadA)	
Mode of measurement	Online SEC-SAXS	
Temperature (°C)	15.0	10.0
Protein buffer	50 mM HEPES pH 8.0, 200 mM NaCl, 1% (v/v) Glycerol	
SEC-Column	Superdex 200 Increase 10/300 GL	Superdex 200 Increase 3.2/300 GL
Injection volume (µl)	100	50
Flowrate (ml/min)	0.5	0.075
Exposure time (# frames)	0.995 s (3000)	2 s (1200)
# frames used for averaging	55	16
Protein concentrations (mg/ml)	6.00	10.00
Substrate	-	400 µM Leader 3
Structural parameters		
*Guinier Analysis (PRIMUS)*		
*I*(0) ± *σ* (cm^−1^)	0.12 ± 0.000066	262.51 ± 0.192
*R*_g_ ± *σ* (nm)	4.48 ± 0.005	4.47 ± 0.005
*s-range* (nm^−1^)	0.091–0.246	0.087–0.288
*min* < *sRg < max limit*	0.41–1.10	0.39–1.28
Data point range	6–62	8–47
Linear fit assessment (*R*^2^)	0.999	0.999
*PDDF/P(r) Analysis (GNOM)*		
*I*(0) ± *σ* (cm^−1^)	0.12 ± 0.000076	260.80 ± 0.165
*R*_g_ ± *σ* (nm)	4.30 ± 0.003	4.41 ± 0.003
*D*_max_ (nm)	13.57	13.78
Porod volume (nm^3^)	439.58	445.04
*s-range* (nm^−1^)	0.188–5.335	0.108–4.670
χ2 / CorMap *P*-value	1.04/0.106	1.09/0.194
Molecular mass (kDa)		
From *I(0)*	n.d.	n.d.
From Qp^4^	272.25	283.46
From MoW2^5^	212.43	264.39
From Vc^6^	250.32	261.37
Bayesian inference^7^	242.63	318.45
From sequence	249.72 (dimer)	253.17 (dimer + substrate)
Shape modeling/software		
Gasbor		
*s-range* (nm^−1^)	0.188–5.332	0.108–4.665
χ2/CorMap *P*-value	1.42/0.0000000001	1.27/0.00000005
SASBDB accession codes^8^	SASDRX6	SASDRY6
Software		
ATSAS Software Version^9,10^	3.0.4	
Primary data reduction	CHROMIXS^11^ / PRIMUS^12^	
Data processing	GNOM^13^	
Ab initio modeling	GASBOR^14^	
Superimposing	SUPCOMB^15^	
Model visualization	PyMOL^16^	

‡s = 4πsin(θ)/λ, 2θ – scattering angle, λ – Xray-wavelength.

*n.d.* not determined.

### Interactions observed in the ligand-bound structure of MadB

For class I modification enzymes such as NisC or NisB, the FD/NLD/N box located in the LP is essential for substrate recognition^5,6^. Therefore, the mutant MadL2 AAAA, which contains a mutation of this motif into four alanine residues, was created and measured (Fig. S6A). As expected for this mutation, no binding was detected in ITC measurements (Fig. S6A and Table S2). Guided by the structure of the complex, the lysine residues (*K*^−10^ and *K*^−12^ of the LP) involved in binding to the RRE (Fig. 5B) were changed to serine residues, resulting in the mutant MadL2 VSVS, and investigated as to which extent these amino acids influenced the affinity of substrate binding. The mutant still manifested an isotherm indicating specific binding (Fig. S6B) with the same stoichiometry as the leader variants MadL1-3 (MadB dimer: substrate—1:1, Table S2). However, the *K*_D_ value increased to 2.49 ± 1.24 µM, which is ~8-fold higher than the one for MadL2, indicating lower affinity. Consequently, the lysine residues are not essential for substrate binding but still have a stabilizing role. The double mutant MadL2 dM, containing both mutations, MadL2 AAAA and MadL2 VSVS, revealed no specific binding to MadB as already described for the MadL2 AAAA mutant (Fig. S6C and Table S2). The nanoDSF measurements also revealed no protein stabilization by all three mutants of MadL2 (Table S1).

### A putative MadBC-MadA complex

**Fig. 6:**
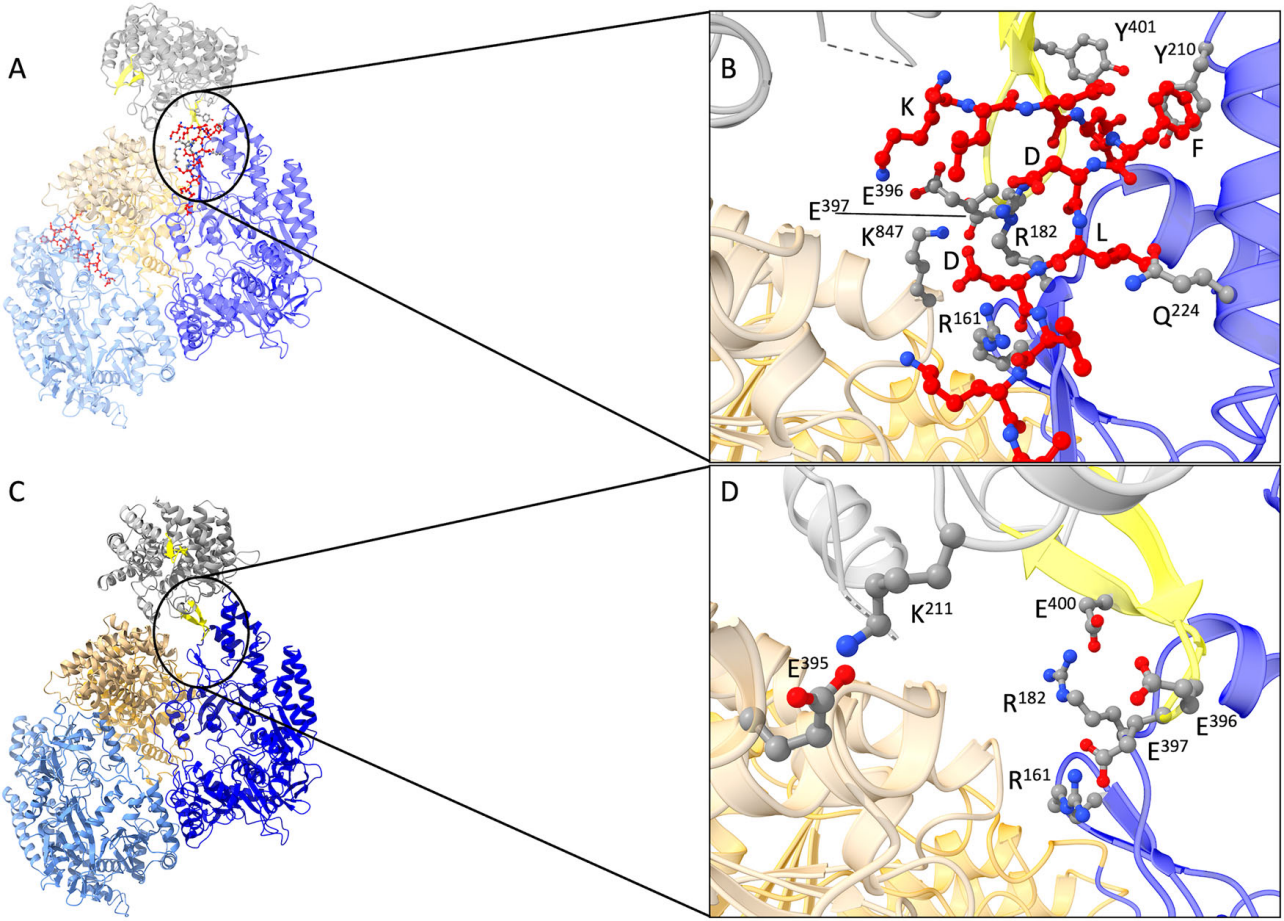
Tentative MadBC complex predicted by protein-protein docking. **A** Predicted complex of MadB and MadC in the presence of MadA including the LP (red ball-and-sticks). Color coding is identical to Figs. 2 and 3. In the zoom-in (**B**), the interactions of E^396^ and E^397^ located in the strand-loop-strand extension of MadC ^18^ with K^847^ of MadB and the N-terminal K of the LP are evident. The orientation of E^396^, E^397^, and Y^401^ of MadC resembles that of the “FDLD” motif of the LP. This prompted a docking of the complex in the absence of the LP, where the two glutamates (in the strand-loop-strand extension of MadC) and the residues in the vicinity of the position of MadA in (**B**) were used as constraints and resulted in the MadBC complex displayed in (**C**). In this proposed complex, MadC is slightly turned with respect to the previous complex. This results in a larger interaction surface between the proteins. In the close-up (**D**), additional salt bridges are evident, particularly of E^397^ and D^400^ of the strand-loop-strand extension of MadC and R^161^ and R^182^ of MadB.

To obtain first insights into the architecture of the MadBC complex, HADDOCK2.4^28^ was used in a two-step process. The recently determined structure of MadC^18^ was docked to the MadL3-bound structure of MadB. To generate constraints for docking, several assumptions were made. First, it was assumed that the catalytic center of MadC should be close to the peptide in the MadBC complex to facilitate its cyclization. Second, as the “FDLD” motif in the LP is important for MadBC complex formation, this motif should take part in the interactions during initial complex formation. Using these constraints to guide the docking resulted in a possible MadBC complex (HADDOCK docking score of –80.2) with expected and unexpected features (Fig. 6). As presumed, the catalytic center of MadC is oriented towards MadB in close proximity to the LP of MadA. Interestingly, residues in the short β-sheet close to the catalytic center, a feature found in LanC^18^, interact with the LP and surrounding residues in MadB (Fig. 6B). Here, E^396^ and E^397^ in the β-sheet of MadC interact with the N-terminal K^−3^ of the LP and K^847^ of MadB, respectively. The LP is located between the β-sheet of MadC and its catalytic center. A striking feature of this interaction is that residues E^396^, E^397^, and Y^401^ are oriented in a way that resembles the “FDLD” motif of the LP (Fig. 6B), so that they might replace this motif in an interaction with MadB and thus free the LP. To elicit the possibility of this interaction for the complex structure, in which the LP is no longer bound to MadB or in which the peptide egresses from MadB into MadC for further modification, the similarity between the “FDLD” motif and the β-sheet in MadC was exploited. Hence, residues in the β-sheet of MadC and in the area surrounding the now-removed peptide in MadB were used as constraints in docking. This resulted in a tentative structure of the MadBC complex without the peptide (HADDOCK docking score of –80.2). Similar to the first complex (Figs. 6C and 4D), the interaction surface between MadB and MadC is increased due to a slight turn of MadC with respect to MadB, resulting in a more favorable HADDOCK score for the complex without the peptide (Fig. 6C) (−80.2 vs. −121.3). As the fulfillment of the constraints of the docking is part of the scoring function^28–30^ and the sets of constraints differ between the two dockings, this is only indicative of a more stable complex. The β-sheet of MadC adopts now more directed interactions with MadB in the complex without the LP (Fig. 6D) so that E^397^ and D^400^ of MadC interact with R^161^ and R^182^ of MadB, respectively. Thus, it replaces the key interactions of the “FDLD” motif.

## Discussion

Structural information of class I LanB enzymes is currently restricted to the full-length dehydratases of the nisin^22^ and NAI-107^21^ systems, namely NisB and MibB, respectively. However, the NisB structure represents the ligand-bound state, while the structure of MibB adopts the apo state. Thus, we aimed to obtain structural and functional information, especially the underlying conformational changes of a dehydratase and focused in this study on MadB, the dehydratase of the recently identified MadA system^1^.

The ITC measurement of the LP mutant MadL2 VSVS revealed an approximately 8-fold increase of the K_D_ value compared to the wild-type leader variant (MadL2: 0.32 ± 0.04 µM; MadL2 VSVS: 2.49 ± 1.24 µM) and demonstrates an important role of these residues in substrate binding (Fig. 3 and Table S2). In the structure of MadB in complex with MadL3, four interactions with the two lysine residues of MadL3 are present. However, only two interactions (one per lysine residue; Asp^785^ – Lys^−12^; Glu^178^ – Lys^−10^) are affected by the mutation as the other two interactions occur with the backbone of the peptide and are very likely not influenced (Fig. 5B). Due to the different size and different charge between lysine and serine, the interactions to the Asp^785^ and Glu^178^ are not possible due to an increased distance between the interaction partners (2.8–6.5 Å for the −12/785 pair and 3.6–4.8 Å for the −10/178 pair), which explains the decrease in binding affinity. The ~8-fold effect on substrate binding only by interrupting two interactions highlights that the leader binding “pocket” of LanB’s can be highly specific.

Besides the importance of the LP, we also analyzed the influence of residues of the CP on affinity. We created three different LP variants of MadA (MadL1-3) as previously mentioned and determined the thermodynamic parameters of binding to MadB by ITC (Table S2). These results highlighted a significant influence of residues of the CP on the binding affinity. MadL2 revealed a higher affinity compared to the LP only (MadL1). This could be explained with the occurrence of the first threonine and serine residues, which are targets of MadB for dehydration. However, the extension to 10 aa of the CP (MadL3) led to an even larger increase in affinity (K_D_ value (MadL1): 0.41 ± 0.04 µM; *K*_D_ value (MadL3): 0.15 ± 0.01 µM), which could only be explained by the additional presence of the first cysteine residue or the second threonine residue. It can be assumed that threonine and serine residues or the whole CP itself will have several interactions with MadB leading to a higher substrate affinity. Due to missing electron density in this area of the substrate in the cryo-EM structure, no further conclusions on possible interactions can be drawn and further investigations are necessary. However, our results are in agreement with previous studies highlighting the influence of the CP^5,31^ and it can be assumed that the specificity towards the CP of lanthipeptides in LanBs deserves a more detailed investigation in future applications. Overall, our analysis further emphasizes the high specificity of LanBs towards the LP as well as towards the CP of their corresponding substrate. The latter one should not be neglected if synthetic lantibiotics are synthesized by the class I modification machinery.

The ITC measurements showed clearly, for all substrates, a stoichiometry of one substrate per MadB dimer. Furthermore, in the SAXS experiments one tunnel in the envelope of MadB was present, which might serve as an area for substrate entrance and binding (Fig. 4C). The sample of MadB incubated with MadL3 (Figs. 4C and S5) emphasizes this hypothesis as the tunnel was closed in the corresponding SAXS measurement. Further support of our notion comes from SPR and SAXS measurements of the nisin system^5,23^. In contrast, the structure of MadB in complex with MadL3 indicated a stoichiometry of one substrate per MadB monomer, which is in agreement with previously determined crystal structures of class I LanBs with its corresponding LanA or substrate analogs^22,24^. However, it needs to be stressed that we imposed C2 symmetry during refinement, implying that this symmetry might cause the artificial 1:1 stoichiometry.

The two structures determined for MadB allowed for the first time to derive the conformational changes occurring in a class I dehydratase upon substrate binding. Much to our surprise, the only significant structural change in a dimeric protein of more than 2000 amino acid residues occurred in a loop preceding the RRE (Fig. 5C, D as well as Movies S1 and S2). Other changes are confined to surface areas of MadB and likely play no significance role in enzyme catalysis. Residues 164–179 rotated by 8°, which is a prerequisite for substrate binding and formation of the RRE as the β-strand of the LP would severely clash with the loop in its position in the apo structure and thereby prevent binding of the LP (Movies S1 and S2) as it acts as a lid closing the pathway for the substrate to bind. This function suggests that this loop acts as the “regulatory loop” controlling substrate binding and subsequent dehydration reactions. In the apo state of MibB the corresponding residues (211–229; Fig. S1) contain an insertion of three residues (221–223) and a large part of the loop, residues 220–228, is disordered and cannot be traced in the structure. However, the remaining part adopts a similar conformation as the loop in the apo-state of MadB. In the NisA-bound structure of NisB, residues 156–169 (Fig. S1) adopt a similar conformation as the regulatory loop of MadB, although the loop is shortened by six residues in NisB. Thus, the current and limited structural information implies that this regulatory loop is present in all class I dehydratases and plays a similar role as in MadB. However, more structures of class I dehydratase in the two conformations are required before solid conclusions can be drawn. Notably, it is obvious that the ligand-bound conformation of the regulatory loop in MadB is the preferred orientation as indicated by the increased number of interactions to which the ligand also contributes.

Loop dynamics in enzyme catalysis are important factors to regulate enzyme activity as it offers an efficient way to conformational plasticity^32–34^. It has been observed in many protein families such as dihydrofolate reductase^35^, triosephosphate isomerase^36^, protein tyrosine phosphatases (PTPs)^37^, and also RiPPs^38^. In the latter case, class A linaridins contains a S-[(Z)-2-aminovinyl-D-cysteine (AviCys) moiety at the C-terminus. This moiety is installed by LinD (linaridin biosynthesis) or LanD (lanthipeptide biosynthesis), flavoproteins that catalyze the oxidative decarboxylation and subsequent ring formation yielding AviCys. In this particular example, the enzyme is CypD. The enzymes contains a substrate binding clamp composed of 11 residues, which is unstructured in the absence of substrate. Its fluctuations change the size of the substrate binding site as it first moves outward to allow substrate binding and subsequently forms a two-stranded β-sheet in the presence of substrate closing the active site and re-opening again for product release^38^. In PTPs, an aspartate residue located in a flexible loop acts as a general acid/base during catalysis. Thus, loop dynamics ensure that the catalytic active residue is in close and proper vicinity in the presence of the substrate. This is obviously not the case for LinD enzymes and MadB. Rather, the substrate shifts the conformational equilibrium towards the substrate-bound state^33^. The fact that the conformation of the regulatory loop in the apo- and substrate-bound states of MadB is well defined suggests that the equilibrium between the two loop conformation is extremely shifted towards the apo state in the absence of substrate and shifted nearly quantitatively towards the substrate-bound conformation. However, further experiments including extensive MD simulations are required to shed further light on the precise mechanism of the dynamics of the regulatory loop.

As shown in Fig. 1, experimental evidence for the nisin system strongly suggests that NisB and NisC form a complex during installation of the PTMs^23,39,40^. Consequently, we modeled a putative complex of MadB and MadC and present here putative complexes of MadBC in the presence and absence of fully unmodified MadA (Fig. 6). While the exact structures of these complexes have not been verified so far and a rearrangement of the complex upon the release of the peptide from MadB is speculative, these complexes offer new perspectives on complex formation and the role of the catalytic center-proximal strand-loop-strand motif^18^ in the families of class I cyclases and class II cyclase-domains. Although this strand-loop-strand motif does not directly interact with the ‘FDLD’ motif in the LP, it replaces key interactions with MadB in the complex in the absence of the peptide. It also binds in an orientation that the LP is positioned between the strand-loop-strand motif and the catalytic center and interacts with the N-terminal residue of the LP. Hence, the LP might prime MadB for the interaction of MadC, as the same arginine residues in MadB are addressed by either the β-sheet or the “FDLD” motif, and initiate binding of the strand-loop-strand motif to its N-terminal lysine. It then might replace the leader in the complex and simultaneously move the leader towards the catalytic center so that the peptide can subsequently be modified. This hypothesis is in line with the experimentally determined directionality (N- to C-terminus) of the nisin modification machinery^41^. Here, alternating dehydration and cyclization must occur to allow the observed directionality. Such a mechanism requires a back-and-forward switching of the maturation complex to enable the individual reactions in a stepwise manner. However, in the MibA system, no such N- to C-directionality rather the opposite direction (C- to N-directionality) was observed^21^. Independent of which directionality occurs in the MadA system or whether the introduction of the PTM reactions is stochastic, stepwise dehydration and cyclization reactions within the MadBC complex would result in the formation of intermediates. Isolation or identification of such intermediates in future studies is truly required to fully understand the catalytic process of dehydration and cyclization. Nevertheless and regardless which mechanism is operational, the predicted complex in the presence of the peptide highlights that the strand-loop-strand motif could play an important role in the formation of the MadBC complex formation (Fig. 6).

In this study, the dehydratase MadB from *C. maddingly* was characterized and its structure in the apo and ligand-bound state was determined by single particle cryo-EM. Our ITC data demonstrated that residues of the CP have a positive effect on complex stability and increase binding affinity. Structural analysis revealed that only a very small conformational change occurred upon ligand binding. This is now the first time that both states of class I lantibiotic dehydratases have been determined for the same protein so that direct visualization of the underlying conformational changes is possible. In MadB, only a loop preceding the RRE had to rotate by 8° to allow binding of the LP, more precisely the region containing the FDLD box of MadA. Without such a rotation of the regulatory loop, severe steric clashes would prevent LP binding. These findings will have implications for the characterization of new lantibiotics or the design and synthesis of synthetic antibiotics.

## Materials and methods

### Cloning of His_6-_MadB

We cloned the *madB* gene via the Gibson assembly method into a pET28b(+)-vector (Novagen, Germany). This construct resulted in a hexahistidine tagged dehydratase (His6-MadB) with a thrombin cleavage site localized in between. The known sequence of MadB (NCBI: EKQ50562.1) was ordered in a pUC57 vector from GenScript (Netherlands).

### Expression of His_6-_MadB

Plasmid pET28b(+)_madB was transformed into E.coli BL21 (DE3) cells (Invitrogen, Germany) and plated on 2YT-Agar plates containing kanamycin (30 µg/mL). The agar plates were incubated overnight. Precultures were prepared using 2YT-media (100 mL; 30 µg/mL kanamycin) with a cryo stock or freshly colonies from 2YT-agar plates. After overnight incubation, the main culture (1 L) was inoculated with the preculture to an OD_600_ of 0.1. The incubation was performed at 37 °C and 180 rpm until the cell culture reached an OD_600_ of ~0.5. Then, the temperature was reduced to 18 °C for the following incubation. At an OD_600_ of 0.8, 1 mM of the inducer IPTG was added to the culture, which was incubated overnight. Afterwards, the cells were harvested at 5000 × *g* for 15 min at 4 °C and subsequently frozen with liquid nitrogen for the storage at −20 °C.

### Purification of His_6_-MadB

Cells were thawed at room temperature and resuspended in 50 mM HEPES, 500 mM NaCl, 10% (v/v) glycerol, pH 8.0 (at 10 °C). The cell lysis was performed with a Microfluidics cell disruptor (IUL, Germany). Afterwards the lysed cells were centrifuged at 4 °C for 60 min at 100,000 × *g* followed by the addition of imidazole (final concentration 20 mM) to the supernatant for the following immobilized metal ion affinity chromatography (IMAC).

The IMAC was performed with a 5 mL HiTrap^TM^ Chelating HP column (Cytiva, Germany), which was equilibrated with 10 mL IMAC Low buffer (50 mM Hepes, 50 mM NaCl, 20 mM Imidazol, pH 8.0 (at 10 °C)) using an ÄKTA purifier system (Cytiva, Germany). Subsequently, the cytoplasmic fraction after cell lysis and centrifugation was loaded on the column with a flow rate of 0.5–0.75 mL/min. After the loading step, the column was washed with 100 mL of the IMAC low buffer at a flow rate of 1 mL/min. MadB was eluted by increasing the imidazole concentration from 20 mM to 150 mM in a gradient over 75 min.

The elution fractions were pooled and concentrated in a 100 kDa cut-off Amicon^TM^ Centricon (Merck, Germany) and the concentrated pool was used for the following size exclusion chromatography (SEC). The column used for SEC was a Superdex 200 Increase 10/300 GL (Cytiva, Germany), which was equilibrated in the respective buffer as indicated. The purity of the MadB sample was analyzed via Coomassie-stained SDS PAGE and Western blot using an α-His antibody (Qiagen, Germany; dilution 1:1000) (Fig. 1).

### SEC-MALS analysis of MadB

To determine the molecular weight and stoichiometry of the presumably cyclase MadC a combination of size exclusion and MALS was used to determine the oligomeric state in solution. The analyses were performed on an Agilent 1260 HPLC System in combination with a triple-angle light scatter detector (miniDAWN TREOS, Wyatt Technology, Europe) and a differential refractive index detector (Optilab rEX, Wyatt Technology Europe).

Analysis of isolated MadB was performed by injection of 100 μL of a 20 μM solution. A volume of 100 μL was applied on a pre-equilibrated (MALS buffer (50 mM HEPES pH 8.0, 200 mM NaCl) Superdex 200 10/300 Increase column (Cytiva, Germany) at a flow rate of 0.5 mL/min. Analysis of the MadB MadL3 complex was performed as described above for apo MadB with the expection that 20 µM MadB and 20 µM MadL3 were incubated for one hour at 4 °C prior to the analysis. Data analysis was performed with the ASTRA software package (Astra V 5.3.4.20) (Wyatt Technology).

### Small-angle X-ray scattering

We collected the SEC-SAXS data of MadB on beamline P12 **(**PETRA III) at the DESY Hamburg^42^, equipped with a PILATUS 6 M detector (Dectris) at a fixed distance of 3.0 m. The measurement of MadB (6.0 mg/ml, 100 µL inject) was performed at 15 °C on a Superdex 200 Increase 10/300 GL column (50 mM HEPES pH 8.0, 200 mM NaCl, 1% (v/v) Glycerol) with a flowrate of 0.5 ml/min, collecting one frame each 0.995 s. Data were scaled to absolute intensity against water. We collected the SEC-SAXS data for MadB preincubated with MadL3 _-23 – 10_, on beamline BM29 at the ESRF Grenoble^43,44^, equipped with a PILATUS 2 M detector (Dectris) at a fixed distance of 2.827 m. The measurement of MadB with MadL3 (10.0 mg/ml, 400 µM MadL3, 50 µL inject) was performed at 10 °C on a Superdex 200 Increase 3.2/300 GL column (50 mM HEPES pH 8.0, 200 mM NaCl, 1% (v/v) Glycerol) with a flowrate of 0.075 ml/min, collecting one frame each 2 s. Data were scaled to absolute intensity against water. All used programs for data processing were part of the ATSAS Software package (Version 3.0.2)^45^. Primary data analysis was performed with the program CHROMIXS^46^ and PRIMUS^47^. With the Guinier approximation^48^, we determine the forward scattering *I(0)* and the radius of gyration (*R*_*g*_). The program GNOM^49^ was used to estimate the maximum particle dimension (*D*_*max*_) with the pair-distribution function *p(r)*. Low resolution ab initio models were calculated with GASBOR^49^ and superimposed with the predicted model with SUPCOMB^50^.

### Isothermal titration calorimetry (ITC)

To investigate the binding parameters between the dehydratase MadB and maddinglicin leader variants ITC was used. To prevent heat resulting due to different buffer mixture, the enzyme and leader variants were separately dialyzed against the ITC buffer (50 mM HEPES, 500 mM NaCl, pH 7.5).

After dialysis, the concentration of the enzyme was determined via Nanodrop and adjusted to 20 µM of the MadB dimer. In contrast, the concentrations of the leader variants were determined via RP-HPLC and afterwards adjusted to a concentration of 200 µM. All experiments were performed using an ITC_200_ (Microcal, Malvern Panalytical, USA). Before the measurement the enzyme MadB was loaded with a volume of around 280 µL into the cell whereas the maddinglicin leader with a volume of 40 µL was used to fill the titrating syringe.

All ITC experiments were performed at 25 °C with 20 injections, in which 2 µL of the maddinglicin leader variant was titrated from the stirring syringe (stirring speed = 750 rpm) into the cell containing the enzyme. The reference power during the measurement was 7 µcal s^−1^ and the spacing time between each injection was 180 s. Each experiment was performed at least in triplicate.

### Differential scanning fluorometry (DSF)

Thermal stability of His6-MadB in presence or absence of ligands was determined by measuring changes in the intrinsic fluorescence using Prometheus NT.48 instrument (NanoTemper Technologies, Germany). The protein was diluted to 2 µM with ITC Buffer (50 mM HEPES, 500 mM NaCl, pH 7.5) and optionally incubated at room temperature for 10 min with the ligand peptides (10 µM, if other not stated). The samples were loaded into “Standard” type glass capillaries and the light excitation power at 280 nm was set to 70%. The temperature ramp of 1 °C/min was applied from 25 to 95 °C while monitoring the intrinsic fluorescence at 330 and 350 nm. The fluorescence intensity ratio, its first derivative and the melting temperature *T*_m_ were calculated using PR. Stability Analysis software (NanoTemper Technologies, Germany).

### Single particle cryogenic-electron microscopy (cryo-EM)

Samples were prepared by applying 3 µL purified sample to glow-discharged (PELCO easiGlow Glow Discharger, Ted Pella Inc., USA) Quantifoil R1.2/1.3 400-mesh copper grids (Quantifoil Micro Tools GmbH, Germany).

The excess liquid was removed by blotting with filter paper for 3 s and grids were immediately vitrified by plunge freezing in liquid ethane at 77 K using a Vitrobot Mark IV (Thermo Fisher Scientific) operated at 100% humidity and 10 °C temperature. Frozen grids were stored at liquid nitrogen temperature before usage. A total of 8100 micrographs for MadB and 8362 micrographs for MadB+MadL3 were recorded on a Talos^TM^ Arctica G2 (Thermo Fisher Scientific), equipped with a Bioquantum K3 (Gatan), operated at 200 kV. Movies were acquired at a nominal underfocus of range of 0.5–3 µm (apo MadB) or 0.5–3 µm (MadB-MadL3), in super resolution mode and dose fractionation (up to 50 frames/movie), with an object pixel size of 0.41945 Å/pixel (apo MadB) or 0.8389 Å/pixel (MadB-MadL3). The accumulated electron dose for each projection was max. 50 e^−^/Å². SerialEM was used for automated data acquisition^51^. Further details of data collection are provided in Figs. S7 and S8, respectively.

### SPA image processing

The image processing workflows followed for each sample are depicted in Supplementary Figs. S7 and S8, respectively. Frame alignment and averaging was done in WARP^52^. All other steps were performed with CryoSPARC v3.1.0^53,54^. In brief, particles were selected in two steps, first using blob detection to create low-resolution models ab initio; these models were then used for a template-based search of particles. Several rounds of 2D and 3D classifications were performed to select more homogeneous particle subsets. Final non-uniform refinements with CTF and defocus refinements were done to improved resolution. The workflow of data collection and processing is summarized in Fig. S7 (apo-MadB) and Fig. S8 (ligand-bound MadB). Since we have no evidence for a tetramer of MadB in solution, we did not include such an oligomer in our discussion and focused only on the dimer of apo MadB and ligand-bound MadB. Further details are provided in Figs. S7 and S8, respectively. Data collection and refinement statistics are provided in Table 2.

**Table 2:** Cryo-EM data collection and refinement statistics

	apoMadB (EMDB-50910) (PDB 9G04)	MadB_MadL3 (EMDB-50911) (PDB 9G05)
Data collection and processing		
Magnification	100,000x	100,000x
Voltage (kV)	200	200
Electron exposure (e^–^/Å^2^)	0.75	1.01
Defocus range (μm)	0.5–2.5	0.5–3
Pixel size (Å)	0.8389	0.8389
Symmetry imposed	C2	C2
Initial particle images (no.)	2,667,864	2,971,174
Final particle images (no.)	800,314	994,872
Map resolution (Å)	2.7	3.13
FSC threshold	0.143	0.143
Map resolution range (Å)	2.3–3.95	2.4–4.5
Refinement		
Initial model used (PDB code)	–	–
Model resolution (Å)	2.9	3.2
FSC threshold	0.5	0.5
Model resolution range (Å)	2.3–3.95	2.4–4.5
Model composition		
Non-hydrogen atoms	34,114	34981
Protein residues	2047	2100
Ligands		2
*B* factors (Å^2^)		
Protein	54.61	40.30
Ligand		
R.m.s. deviations		
Bond lengths (Å)	0.005	0.005
Bond angles (°)	0.773	0.580
Validation		
MolProbity score	1.19	1.44
Clashscore	1.35	2.57
Poor rotamers (%)	0.57	1.01
Ramachandran plot		
Favored (%)	95.3	94.2
Allowed (%)	3.7	5.3
Disallowed (%)	1	0.5

### Docking of the MadBC complex

In order to predict the atomistic structure of the MadBC complex, we utilized HADDOCK2.4^55^ with two different sets of constraints. One for the complex with the MadL3, including the LP, emerging from MadB and one for the complex without a peptide. The default settings for HADDOCK2.4 were used. We exploited the experimentally available information including interacting residues and residues vital for the function of the complex as constraints for HADDOCK2.4. After removing water and cosolvents in MadC we defined surface residues within 8 Å of the catalytic zinc as active residues in HADDOCK. These residues are S50, H51, I^154^, H^228^, E^393^, and D^409^ of MadC. This was done to ensure that the catalytic center of MadC is close to the peptide emerging from MadB. For MadB, we removed co-solvents from the structure, either kept or removed the peptide, and defined two different sets of constraints. First, we used the sequence “FDLD” in the LP as active residues and defined the surrounding residues as passive residues. Second, for MadB without the LP we defined the residues surrounding the previous peptide R^161^, N^173^-R^182^, T^184^-P^186^, I^213^, K^218^, F^222^, Q^225^, L^226^, R^229^, S^781^, N^782^, and R^893^ and for MadC residue E396, E^397^, and G^398^ in the β-sheet as active residues for the docking. The cluster representatives of the clusters with the most favorable score were used for further analysis. Docking scores are provided in the results section.

### Figure preparation

For figure preparation the following programs were used: PRISM 8 (GraphPad) Version 8.0.2, PEAQ analysis software (Malvern Panalytical) Version 1.41, Powerpoint (Microsoft Office) Version 16.43, Adobe Acrobat Pro Version 11.0.23.

Figures of the structures and the AlphaFold2^56^ models were prepared using the PyMol software suite (www.pymol.org)^57^ and ChimeraX^58^.

### Statistics and reproducibility

All ITC results were obtained from at least three independent biological replicates to confirm reproducibility. Data are shown for single experiments without errors. Evaluation of the data in Tables S2 is shown as mean ± SD. The number of biological replicates (N) is detailed in the figure legends. Statistical analyses were performed using GraphPad Prism (version 10.4.1 for MacOS, GraphPad Software, Boston, Massachusetts, USA).

### Reporting summary

Further information on research design is available in the Nature Portfolio Reporting Summary linked to this article.

## Supplementary information

Supplementary Information

Movie S1

Movie S2

Movie S3

Supplementary Data 1

Supplementary Data 2

Description of Additional Supplementary Files

Reporting summary

## Data Availability

All data generated or analyzed during this study are included in this published article (and in its accompanying Supplementary Information). We uploaded the SAXS data to the Small Angle Scattering Biological Data Bank (SASBDB)^59,60^, with the accession code SASDRX6 (apo-MadB, https://www.sasbdb.org/data/SASDRX6) and SASDRY6 (MadB_MadL3, https://www.sasbdb.org/data/SASDRY6). The atomic coordinates and structure factors have been deposited in the Worldwide Protein Data Bank (PDB) (https://www.wwpdb.org/) under the following accession code for MadB (PDB: 9G04 and EMDB 50910) and MadB-MadL3 complex (PDB: 9G05 and EMDB 50911). Raw data of the SEC-MALS, nanoDSF, and ITC measurements are available as Supplementary Data 1 and 2, respectively.
