# Supplementary material for: Structural insights into the substrate binding mechanism of the class I dehydratase MadB
Source: Commun Biol. 2025 Jul 9;8:1032. doi: 10.1038/s42003-025-08454-5 (PMC12241602; doi:10.1038/s42003-025-08454-5)
Supplement: Supplementary file 1 — Supplementary Information [file 42003_2025_8454_MOESM1_ESM.pdf]

Supplementary Information

Table S1: NanoDSF Data of MadB dimer and the LP variants. For experimental details, see Materials and Methods.

Sample	T_m [°C]	Δ
MadB	48.36	-
length variants of LP		
MadB + MadL1	50.16	+ 1.80
MadB + MadL2	50.48	+ 2.12
MadB + MadL3	51.18	+ 2.82
mutation variants of LP		
MadB + MadL2_AAAA	48.31	- 0.05
MadB + MadL2_VSVS	48.18	- 0.18
MadB + MadL2_dM	48.32	- 0.04

Table S2: ITC Data of MadB dimer and the LP variants. All experiments were performed at least in triplicate, and the error represents the standard deviation (S.D.) of a minimum of three independent experiments. For experimental details, see Materials and Methods.

Maddinglicin LP variants	N	Kd *[µM]*	Δ H *[kJ/mol]*	-TΔ S *[kJ/mol]*	Δ G *[kJ/mol]*
MadL1 $_{(-23 – -1)}$	1.21 ± 0.01	0.41 ± 0.04	-57.36 ± 1.26	19.15 ± 5.01	-36.68 ± 0.88
MadL2 $_{(-23 – +5)}$	0.89 ± 0.01	0.32 ± 0.04	-74.28 ± 6.96	23.54 ± 5.49	-36.10 ± 1.26
MadL3 $_{(-23 – +10)}$	0.97 ± 0.01	0.15 ± 0.01	-61.35 ± 0.92	23.56 ± 10.69	-37.83 ± 1.65
MadL2_VSVS	1.12 ± 0.14	2.49 ± 1.24	-56.93 ± 1.22	20.26 ± 5.49	-36.67 ± 1.08
MadL2_AAAA	No binding observed in ITC measurements				
MadL2_dM	No binding observed in ITC Measurements				

Figure S1

```
MadB      MRDLYR-NTNTFMIRTPIFSID-------------NYYEF--------------------   26
NisB      MIKSSF-KAQPFLVRNTILCPN-------------DKRSF--------------------   26
MibB      MTDSPFRAWDVFMVRTPVGYAYPTPLSNSGFDSPASSPGFGEGEFPPDAPVPSDVSGHGA   60

MadB      ------FRKDG--ESDKIKDRLLEICNNSVFREAILVSSKSLYSTIIDFC---DGKE-IK   74
NisB      ---------------TEYTQVIETVSKNKVFLEQLLLANPKLYDVMQKYN---AGLLKKK   68
MibB      GSSEASVRASGRPPAGDHLSLLRAACEDGPLMEAVELASPSLAGLLARVARGDTGGLKDK   120

MadB      KFDYFLQSIYKYLIRMSMRPTPFGLFSGVDFGKYAEETVISYENDNFKKFARPDLEWIIK   134
NisB      RVKKLFESIYKYYKRSYLRSTPFGLFSETSIGVFSKSSQYKLMGK-TTKGIRLDTQWLIR   127
MibB      RLRRAALALLRYDIRMRTRPTPFGLFAGVSGGRFDTSAKWL-AGTGHRTRTRADMEWLLS   179

MadB      IVKELEDNHYKNL--TFKINDSIFIKGERALLIHSTDKEDN---NRIGEISIRATKPFMR   189
NisB      LVHKMEVDFSKKL--SFTRNNANYKFGDRVFQVYTINSSE------LEEVNIKYTNVYQI   179
MibB      AVHRLERDRVLLAGVTVQAHQTLTVRGDRIVLDCPSALGKPLNGSTRSTVSARRSPVVAE   239

MadB      TYDLAKD-GIEYNKLKYILIDEYSIEDESKIDNFLKQLIEREFLISNLRPPLTVLDQFDY   248
NisB      ISEFCENDYQKYEDICETVTLCYGDEYRELSEQYLGSLIVNHYLISNLQKDLLSDFSWNT   239
MibB      ILGAARR-PVLAGRLAQSVAQRFEL-PADRVTGLLADMAAQELLITALRPPLDGDDPLQH   297

MadB      LINEVKKAEI------------EIPLVDELTEIKEKLKLYNETPVGAGEETYLELYKKME   296
NisB      FLTKVEAIDE------------DKKYIIPLKKVQKFIQEYSEIEIGEGIEKLKEIYQEMS   287
MibB      VLDVVAAAEARAGSPAEAMSSESAALVAALREVDARCHAYDRTAVGQGRRELAELIQSTR   357

MadB      SVANVKNILQVDMKLNLRDKKINKKIISDVNDLMNILLDLSMSIENPEPFLSKYKQEFIE   356
NisB      QILENDNYIQIDLISDSEI-NFDVKQKQQLEHLAEFLGN--TTKSVRRTYLDDYKDKFIE   344
MibB      RVHPHDTPLHVDLRIDLEVR-LPEVVRTEIERAAEALWRLSPPRR-GMRALRRYHEAFLE   415

MadB      KYGQDREISLLEMLDNDIGIGPPMNYERPRNNRSLDVSVNELLDNNVRDYFMEKYFQALK   416
NisB      KYGVDQEVQITELFDSTFGIGAPYNYNHPRNDFYESEPSTLYYSEEEREKYLSMYVEAVK   404
MibB      RYGADRAVPLLELLDDTRGLGPPAGYKWPPSETPAGPQEEP----RRSAALARLVAKAAR   471

MadB      TNSRNIAIRDDEIKNLELQKIDYENIPDSLEINLLVKNKSEDNL-SDEFQYYIGPNLGST   475
NisB      NHN-VINLDDL---ESHYQKMDLEKKSELQGLELFLNLA---KE-YEKDIFILGDIVGNN   456
MibB      RGEREIVIDEETIAELAYDEAAPADLPNSLELGVHVVAPSLDELSAGTFRVVLAPGPGSH   531

MadB      SAGKSFGRFSHMMSEPKKFFEELDERNIELIDSEEYVTCEISYLPSEVRNANVTRNIHSS   535
NisB      NLGGASGRFSALSPELTSYHRTIVDSVERENENKEITSCEIVFLPENIRHANVMHTSIMR   516
MibB      HAGATLGRFTGLLPDVDAESA--ARQAGRPLHIQDAVAADVAFIPRSGRAANLAHTPSYS   589

MadB      EYEMSLFTNGSKDNLYRIKLNDIYIGLEN-NTFYAKSKTLNKKLLLTINNM-LNPQTAPN   593
NisB      RKVLPFFTSTSHN---EVLLTNIYIGIDEKEKFYARDISTQEVLKFYITSM-YNKTLFSN   572
MibB      GRRISVGLPDSG-RAQEIPLDELGVGANLERLCLV-HLPTGREVVPALPNMVSAFAQAPN   647

MadB      AIRFLNDISLDEKKLWYKFVWSDVYKDFSYIPAIKYKNFVIMPETWKMNKINMKINKKTE   653
NisB      ELRFLYEISLDDK--FGNLPWELIYRDFDYIPRLVFDEIVISPAKWKIWGRDVNSK--MT   628
MibB      PARLLFELGLEGQRLWEPWDWGAL-SEMPFLPGVRYGRTLLAAPLWRMDQLRGPADDSGP   706

MadB      FNEFKNQFNDYRIKYGVPQYVYITFADNRILLNLDDEQCVKILYHECKNSF---NEIILN   710
NisB      IR-------ELIQSKEIPKEFYIVNGDNKVYLSQKNPLDMEILESAIKKSSKRKDFIELQ   681
MibB      AADWDAALDRWRAEWNVPRRVLAVSMDQRLLLDLDDAWHRVLLRDELRRT----PELIAQ   762

MadB      SYEEEGVNIVKE---SHKDYICELVIPLTKIKQETISDKVSARMLSSDIS-SLSKERVKD   766
NisB      EYF-EDENIINK---GEKGRVADVVVPFIRTRALGNE----GRAFIREKR-VSVERREKL   732
MibB      QVAGDEEGWLDRGDAGFPGHLAEIVVPLERRDRHAARPP--------HIRATVSGREPTG   814

MadB      PFDEWLYIKLYGISSNVDDLIAYYISEFCNELVEEEIISKYFFMRYVDP-EQHIRLRLNS   825
NisB      PFNEWLYLKLYISINRQNEFLLSYLPDIQK--IVANLGGNLFFLRYTDP-KPHIRLRIKC   789
MibB      AGGPWLYLRLRVPRRNQDDFLRDQVPVLVRAGIE-HGADRWFFIRYSDTAGQHLRVRFRG   873

MadB      SQEKLLMI-YPKIREWLSMIRKKGLMTYFSIDSYDREIERYGGIELINIAEKVFFFDSIV   884
NisB      S--DLFLA-YGSILEILKRSRKNRIMSTFDISIYDQEVERYGGFDTLELSEAIFCADSKI   846
MibB      EREKLWAGLLPEIGARLVEWQRQGLLAGHELGQYDPEYERYGGDALAEFTETAFQHDSAA   933

MadB      TEDILRAKREGSFDFCDEIIGMISVVHYMESFGLPYAKQV--------------------   924
NisB      IPNLLTLIKDTNNDWKVDDVSILVNYLYLKCFFQNDNKKI--------------------   886
MibB      AISLLRLTRRAGFRYTLDEVTAISAAALADAFGPPAPVVEPVPLVGGLQWAPDLFDGDPA   993
```

```
MadB      -EFLRSQVSSSEYREDFKQKRTEYMKLCNSNKDWEGLRESEEGNILIEILNKRRKIIEYY   983
NisB      -LNFLNLVSTKKVKENVNEKIEHYLKLLKVNNLGDQIFYDK------NFKELKHAIKNLF   939
MibB      AAWMSSTGGRRELPPDYRRDPARWQKLIDPTGGWPLLRADEDGCQVLAALESRDEAVRRF   1053

MadB      GNKVRENE----EVSTDLSILDSIIHLNCNRMFGIDREFEKKVRALASHALYA----LKH   1035
NisB      LKMIAQDF----ELQKVYSIIDSIIHVHNNRLIGIERDKEKLIYYTLQR-LFV----SEE   990
MibB      GTAYREAFRPTDSPSTQLRLVGSLLHMTCNRLIGGSAERERSVLGLARGAVQDNLNRRRH   1113

MadB      FKS1038
NisB      YMK993
MibB      LA-1115
```

Sequence alignment of MadB, NisB, and MibB created with Clustal Omega . Residues of the glutamylation domain (1- 741) of MadB are highlighted in blue and residues of the elimination domain (742- 1038) in cyan. Residues essential for reactivity of NisB are underlined and highlighted in all three proteins. The regulatory loop of MadB (residues 164 – 179) is underlined.

Figure S2

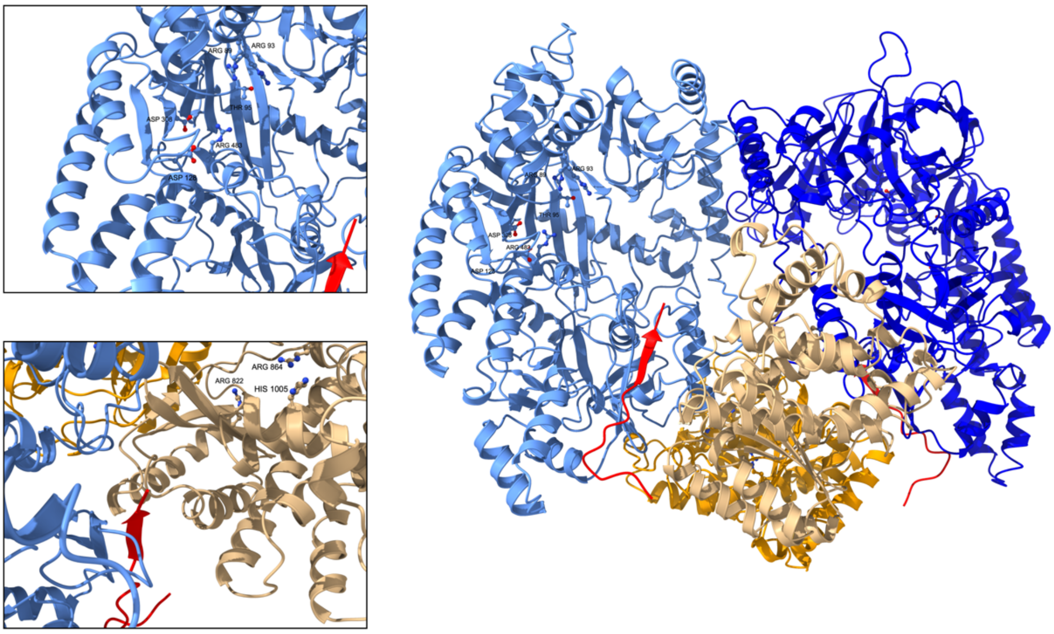

Overall structure of MadB in complex with MadL3 (right panel). Color-coding is as in Figure 2A. Upper left panel: zoom-in into glutamylation domain 1. Residues (Arg^{89}, Arg^{93}, Thr^{95}, Asp^{128}, Asp^{308}, Arg^{483}) that are essential for transfer of Glu from tRNAGlu to NisA are shown in ball-and-sticks representation and labeled. Lower left panel: zoom-in into the elimination domain 1. The three residues (Arg^{822}, Arg^{864}, His^{1005}) that are essential for elimination of Glu from NisA are shown in ball-and-sticks representation and labeled

Figure S3

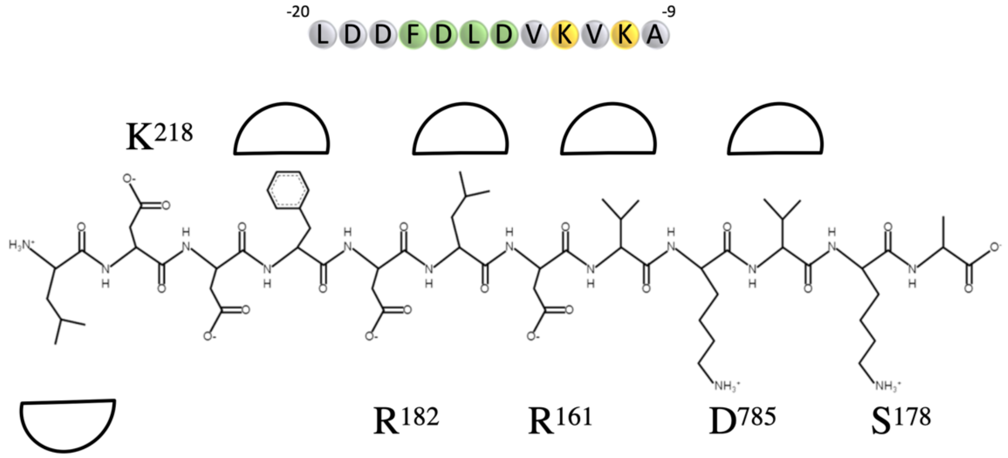

Schematic summary of the interactions of the LP of MadL3 as determined in the ligand-bound structure of MadB. The sequence build into the electron density is show above the scheme and color-coded as in Figure 1D. Polar and charged residues of MadB interaction with the LP are highlighted in one letter code, while hydrophobic interactions are indicated by half circles.

Figure S4

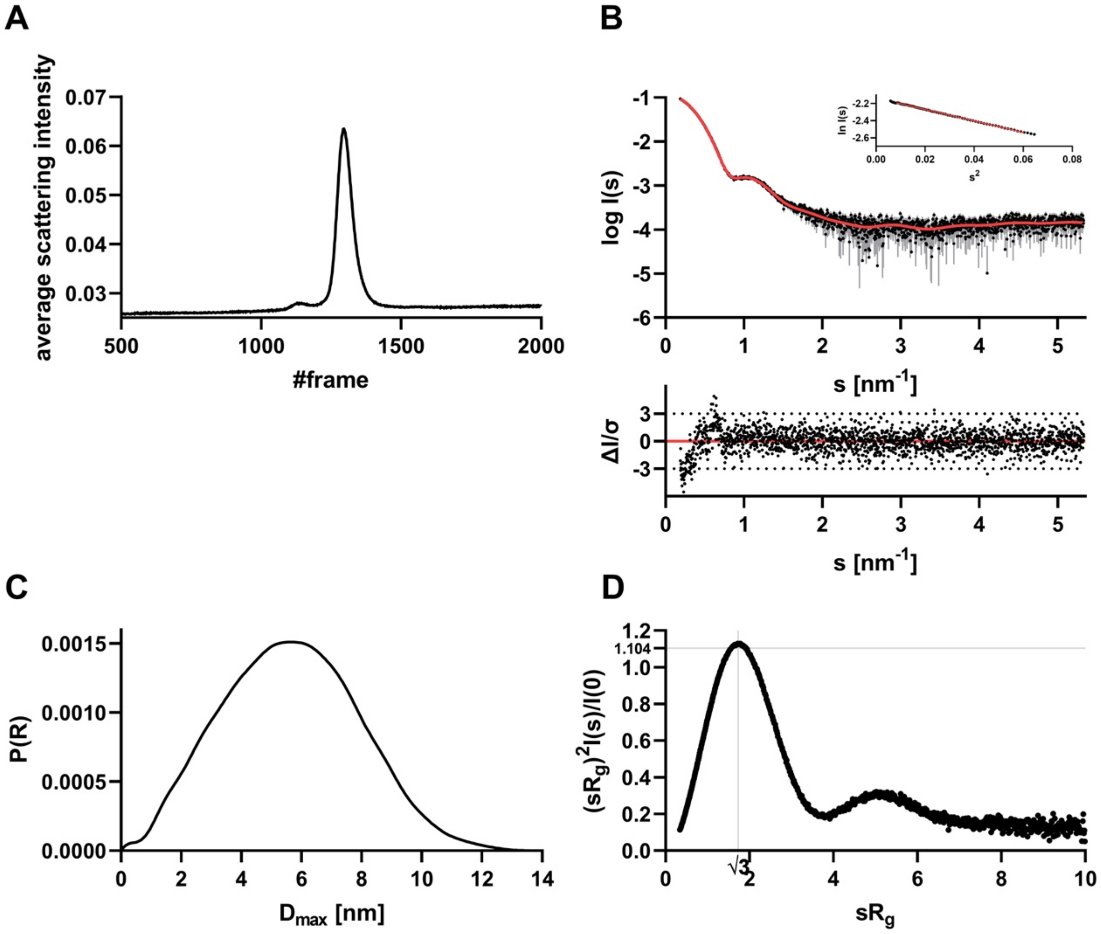

Small-angle X-ray scattering data from MadB apo. (A) SEC SAXS scattering data (from CHROMIXS). **(**B) Experimental data curve is shown in black dots with grey error bars. The GASBOR ab-initio model fit (χ2 of 1.42) as red line and below is the residual plot of the data. The intensity is displayed as a function of momentum transfer s. The Guinier plot of MadB apo is added in the right corner. A stable Guinier region (red line) was found in the range of sR_g < 1.3, with a R_g of 4.48 nm. (C) The Distance distribution, shown as the *p(r)* function offered a maximum particle diameter (D_{max}) of 13.57 nm. (D) Dimensionless Kratky plot showed a compact globular particle.

Figure S5:

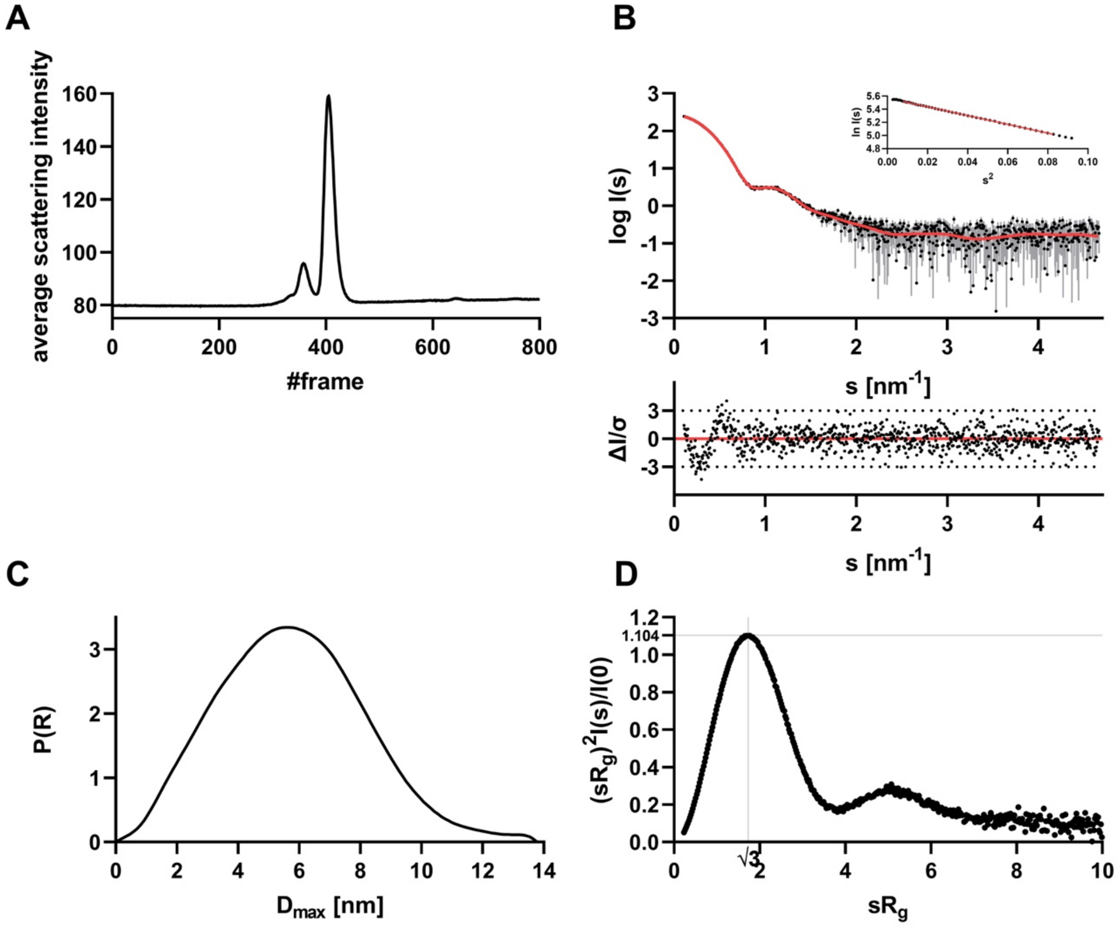

Small-angle X-ray scattering data from MadB with MadL3. (A**)** SEC SAXS scattering data (from CHROMIXS). **(**B) Experimental data curve is shown in black dots with grey error bars. The GASBOR ab-initio model fit ($\chi 2$ of 1.27) as red line and below is the residual plot of the data. The intensity is displayed as a function of momentum transfer s. The Guinier plot of MadB with MadL3 is added in the right corner. A stable Guinier region (red line) was found in the range of $sR_g < 1.3$, with a R_g of 4.47 nm. (C) The Distance distribution, shown as the *p(r)* function offered a maximum particle diameter (D_{max}) of 13.78 nm. (D) Dimensionless Kratky plot showed a compact globular particle.

Figure S6

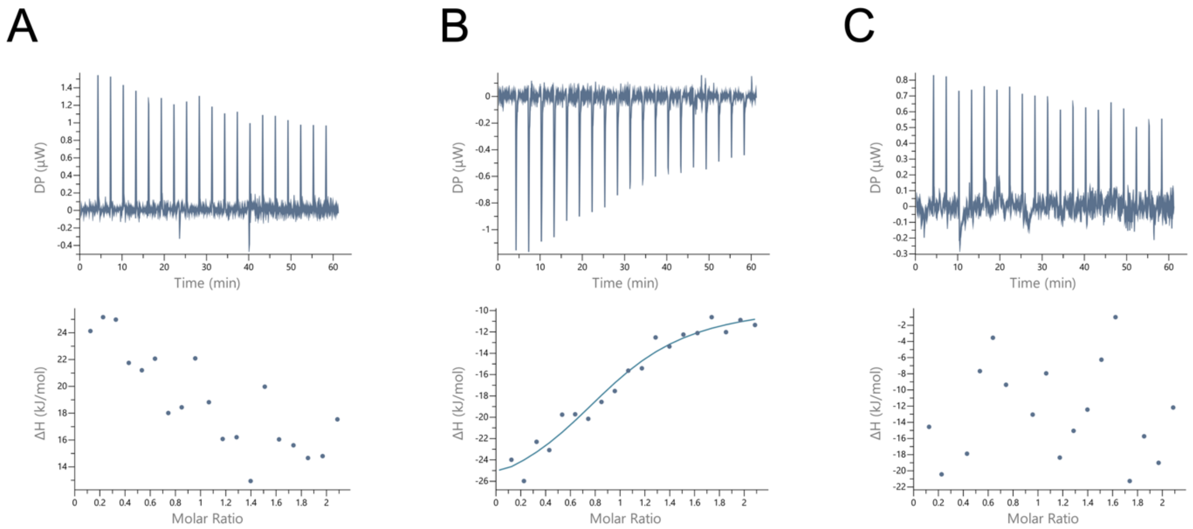

(A – C) ITC raw data (upper panels) and evaluated data (lower panels) are shown for MadL2_AAAA / MadB (A), MadL2_VSVS / MadB (B) and MadL_dM / MadB (C).

Figure S7 Cryo-EM processing workflow for apo-MadB.

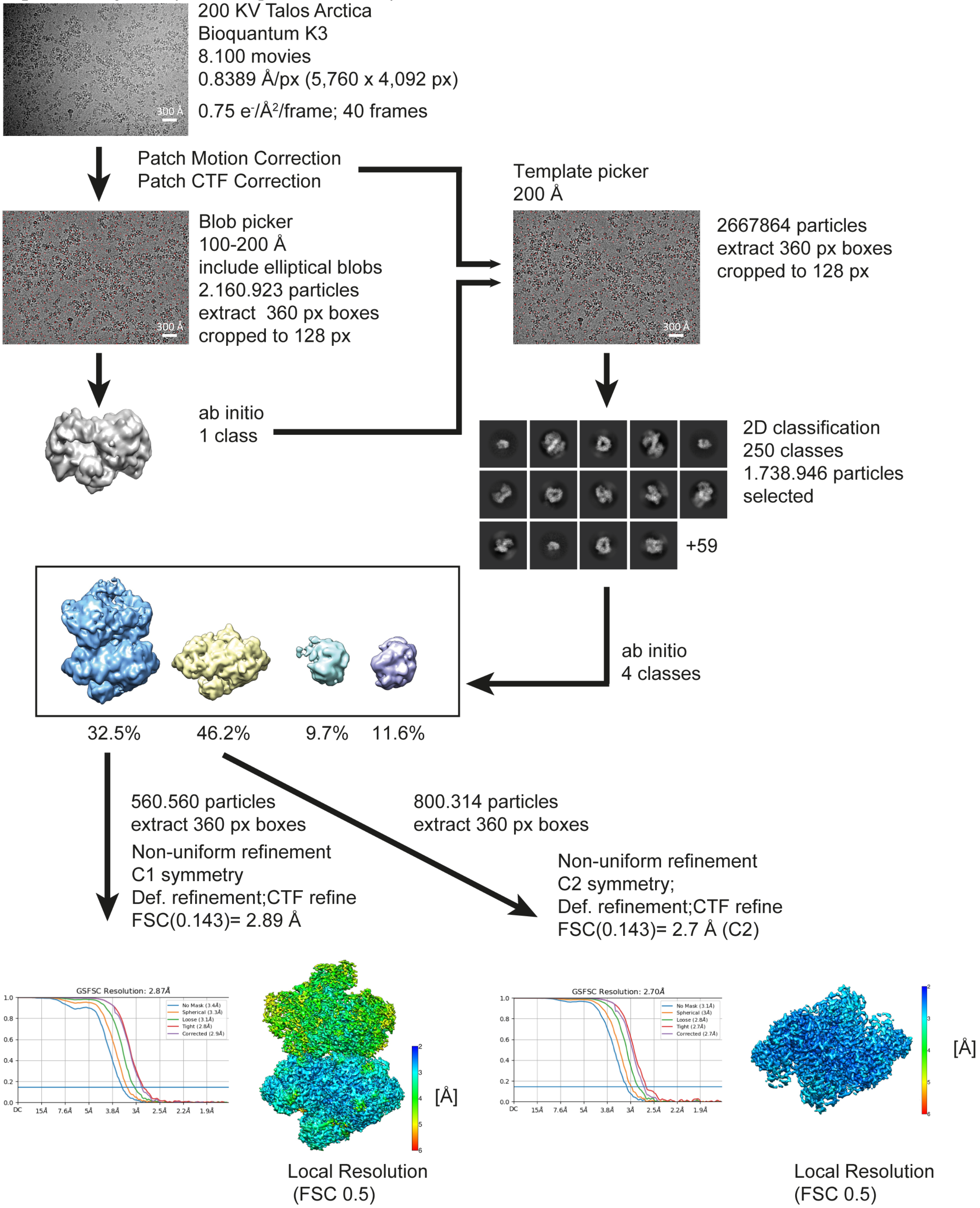

Figure S8 Cryo-EM processing workflow for MadB-MadL3.

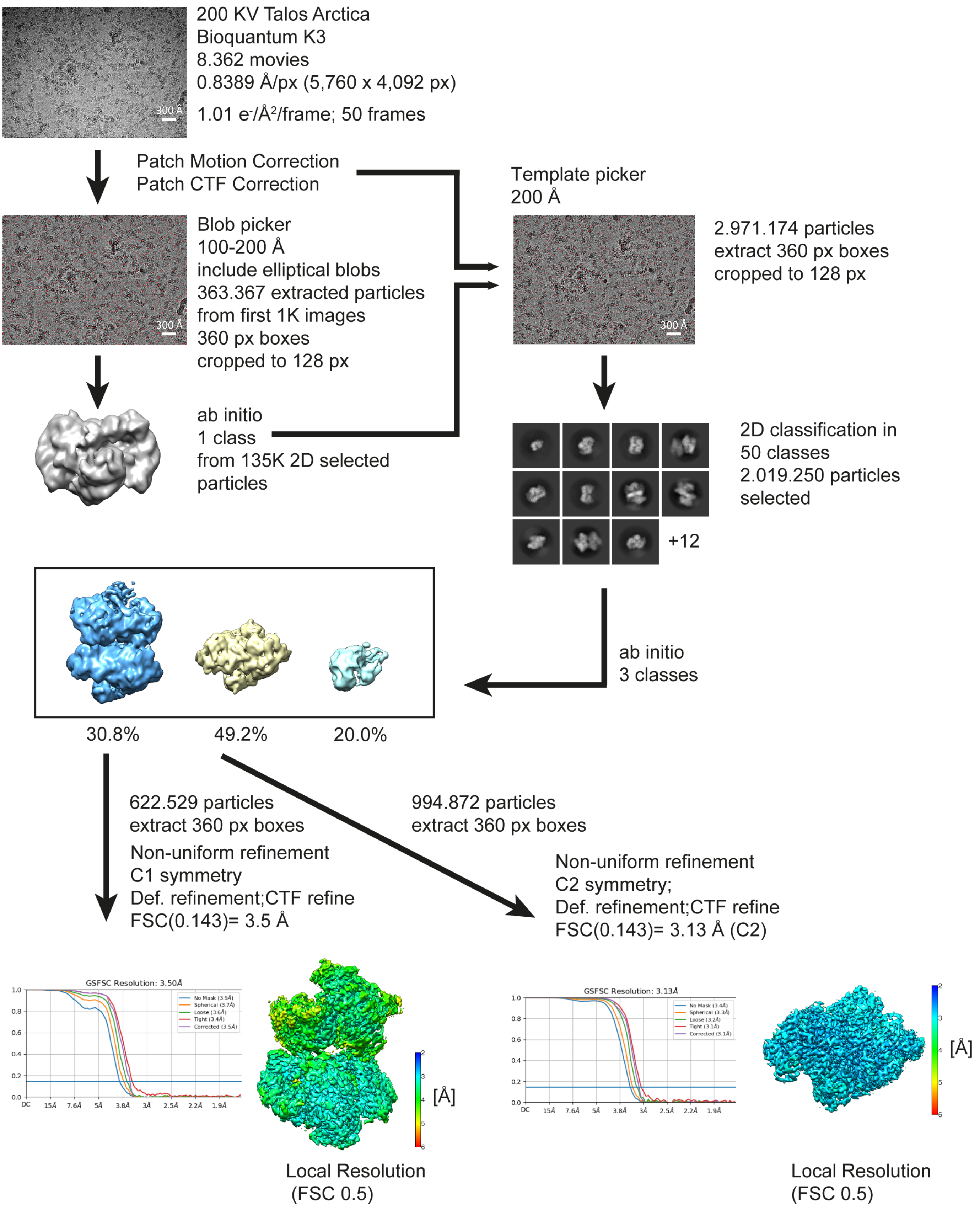

Figure S9 Un-cropped SDS-PAGE of the SDS PAGE shown in Figure 2A.

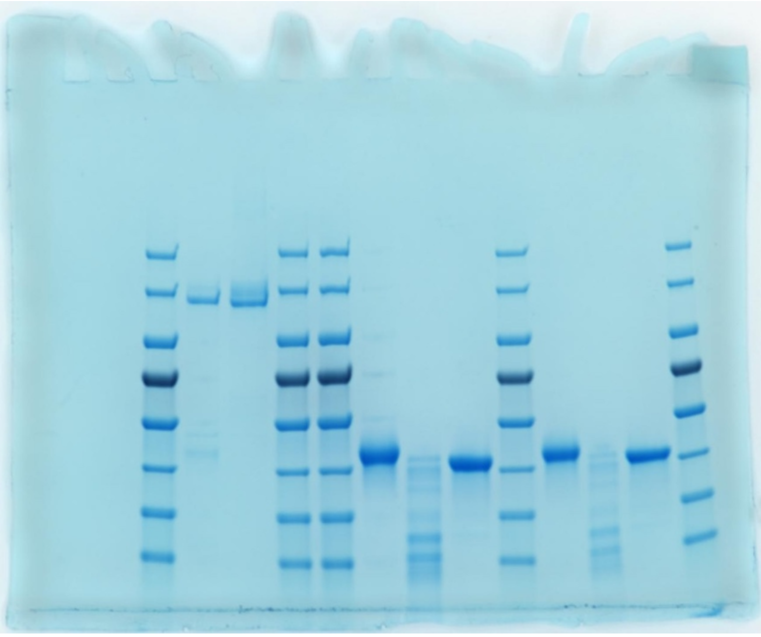

References

1 Blanchet, C. E. *et al.* Versatile sample environments and automation for biological solution X-ray scattering experiments at the P12 beamline (PETRA III, DESY). *J Appl Crystallogr* **48**, 431-443, doi:10.1107/S160057671500254X (2015).

2 Pernot, P. *et al.* New beamline dedicated to solution scattering from biological macromolecules at the ESRF. *Journal of Physics: Conference Series* **247**, 012009 (2010).

3 Pernot, P. *et al.* Upgraded ESRF BM29 beamline for SAXS on macromolecules in solution. *J Synchrotron Radiat* **20**, 660-664, doi:10.1107/S0909049513010431 (2013).

4 Porod, G. Die Röntgenkleinwinkelstreuung Von Dichtgepackten Kolloiden Systemen - 1 Teil. *Kolloid-Zeitschrift and Zeitschrift Fur Polymere* **124**, 83-114, doi:Doi 10.1007/Bf01512792 (1951).

5 Fischer, H., Neto, M. D., Napolitano, H. B., Polikarpov, I. & Craievich, A. F. Determination of the molecular weight of proteins in solution from a single small-angle X-ray scattering measurement on a relative scale. *Journal of Applied Crystallography* **43**, 101-109, doi:10.1107/S0021889809043076 (2010).

6 Rambo, R. P. & Tainer, J. A. Accurate assessment of mass, models and resolution by small-angle scattering. *Nature* **496**, 477-481, doi:10.1038/nature12070 (2013).

7 Hajizadeh, N. R., Franke, D., Jeffries, C. M. & Svergun, D. I. Consensus Bayesian assessment of protein molecular mass from solution X-ray scattering data. *Sci Rep* **8**, 7204, doi:10.1038/s41598-018-25355-2 (2018).

8 Kikhney, A. G., Borges, C. R., Molodenskiy, D. S., Jeffries, C. M. & Svergun, D. I. SASBDB: Towards an automatically curated and validated repository for biological scattering data. *Protein Sci* **29**, 66-75, doi:10.1002/pro.3731 (2020).

9 Manalastas-Cantos, K. *et al.* ATSAS 3.0: expanded functionality and new tools for small-angle scattering data analysis. *Journal of Applied Crystallography* **54**, doi:doi:10.1107/S1600576720013412 (2021).

10 Franke, D. *et al.* ATSAS 2.8: a comprehensive data analysis suite for small-angle scattering from macromolecular solutions. *J Appl Crystallogr* **50**, 1212-1225, doi:10.1107/S1600576717007786 (2017).

11 Panjkovich, A. & Svergun, D. I. CHROMIXS: automatic and interactive analysis of chromatography-coupled small angle X-ray scattering data. *Bioinformatics*, doi:10.1093/bioinformatics/btx846 (2017).

12 Konarev, P. V., Volkov, V. V., Sokolova, A. V., Koch, M. H. J. & Svergun, D. I. PRIMUS: a Windows PC-based system for small-angle scattering data analysis. *Journal of Applied Crystallography* **36**, 1277-1282, doi:10.1107/S0021889803012779 (2003).

13 Svergun, D. I. Determination of the Regularization Parameter in Indirect-Transform Methods Using Perceptual Criteria. *Journal of Applied Crystallography* **25**, 495-503, doi:Doi 10.1107/S0021889892001663 (1992).

14 Svergun, D. I., Petoukhov, M. V. & Koch, M. H. Determination of domain structure of proteins from X-ray solution scattering. *Biophys J* **80**, 2946-2953, doi:10.1016/S0006-3495(01)76260-1 (2001).

15 Kozin, M. B. & Svergun, D. I. Automated matching of high- and low-resolution structural models. *Journal of Applied Crystallography* **34**, 33-41, doi:Doi 10.1107/S0021889800014126 (2001).

16 PyMOL. The PyMOL Molecular Graphics System, Version 2.5 Schrödinger, LLC. (2022).
