# Supplementary material for: Structural insights into the substrate binding mechanism of the class I dehydratase MadB
Source: Commun Biol. 2025 Jul 9;8:1032. doi: 10.1038/s42003-025-08454-5 (PMC12241602; doi:10.1038/s42003-025-08454-5)
Supplement: Supplementary file 7 — Description of Additional Supplementary Files [file 42003_2025_8454_MOESM7_ESM.pdf]

Description of Additional Supplementary Files

File name: Movie S1
Description: Morph from the apo to the ligand-bound state of MadB

File name: Movie S2
Description: Zoom-in morph from the apo to the ligand-bound state of MadB

File name: Movie S3
Description: Electron density of MadL3 bound to MadB

File name: Supplementary Data 1
Description: Raw data figure 1

File name: Supplementary Data 2
Description: Raw data Figure 2
